# Identification of Endoplasmic Reticulum Stress-Related Biomarkers of Periodontitis Based on Machine Learning: A Bioinformatics Analysis

**DOI:** 10.1155/2022/8611755

**Published:** 2022-08-29

**Authors:** Qingyu Zhang, Yuheng Jiao, Ning Ma, Li Zhang, Yuqi Song

**Affiliations:** ^1^Hospital of Stomatology, Jilin University, Changchun 130021, China; ^2^Renji Hospital, School of Medicine, Shanghai Jiao Tong University, Shanghai 200127, China; ^3^The First Hospital of Jilin University, Changchun 130000, China

## Abstract

**Objective:**

To screen for potential endoplasmic reticulum stress- (ERS-) related biomarkers of periodontitis using machine learning methods and explore their relationship with immune cells.

**Methods:**

Three datasets of periodontitis (GSE10334, GES16134, and GES23586) were obtained from the Gene Expression Omnibus (GEO), and the samples were randomly assigned to the training set or the validation set. ERS-related differentially expressed genes (DEGs) between periodontitis and healthy periodontal tissues were screened and analyzed for GO, KEGG, and DO enrichment. Key DEGs were screened by two machine learning algorithms, LASSO regression and support vector machine-recursive feature elimination (SVM-RFE); then, the potential biomarkers were identified through validation. The infiltration of immune cells of periodontitis was calculated using the CIBERSORT algorithm, and the correlation between immune cells and potential biomarkers was specifically analyzed through the Spearman method.

**Results:**

We obtained 36 ERS-related DEGs of periodontitis from the training set, from which 11 key DEGs were screened by further machine learning. SERPINA1, ERLEC1, and VWF showed high diagnostic values (AUC > 0.85), so they were considered as potential biomarkers for periodontitis. According to the results of the immune cell infiltration analysis, these three potential biomarkers showed marked correlations with plasma cells, neutrophils, resting dendritic cells, resting mast cells, and follicular helper T cells.

**Conclusions:**

Three ERS-related genes, SERPINA1, ERLEC1, and VWF, showed valuable biomarker potential for periodontitis, which provide a target base for future studies on early diagnosis and treatment of periodontitis.

## 1. Introduction

Periodontitis is an inflammatory disease caused by bacteria [1]. As a global health care problem, it affects approximately 50% of the world's population, of which 1.1 billion people suffer from severe periodontitis [2]. Typical clinical symptoms of periodontitis include alveolar bone loss, clinical attachment loss (CAL), and periodontal pocket formation, which, if not treated promptly, may lead to pain, aesthetic concerns, progressive tooth loosening, and eventually tooth loss [3]. Currently, periodontitis diagnosis relies mainly on clinical parameters such as bleeding on probing (BOP), probing pocket depth (PPD), CAL, and radiological examinations [4], but these modalities have a certain lag.

Endoplasmic reticulum stress (ERS) is a cellular response that can activate the unfolded protein response (UPR) pathway. As a protective response, accumulation of misfolded and unfolded proteins in cells can induce ERS, which removes misfolded proteins to restore proteostasis, but it can also trigger cell death when overloaded with stress [5]. In addition, ERS can trigger an immune response and inflammation through the crosstalk signaling pathways [6, 7]. It has been found that ERS is closely associated with many conditions, such as cancer, allergic diseases, and cardiovascular diseases, and has brought new diagnostic and treatment strategies [8–10].

The initiation and progression of periodontitis are closely related to multiple factors, but the exact underlying mechanisms remain unclear, especially at the molecular level. However, bacteria and their metabolites and the immune-inflammatory response of the body have been shown to play a joint role in that mechanism [11]. A study has shown that the exposure of human gingival cells to high glucose could increase collagen synthesis and secretion by inducing an ERS response, which corroborated the involvement of ERS in periodontal soft tissue damage [12]. A correlation between ERS and bone metabolism was also found, as UPR was involved in the destruction of alveolar bone in experimental periodontitis in mice [13]. In addition, studies have proven that upregulated ERS in periodontitis participates in the immune response process [14, 15]. The above evidence suggested that ERS was related to the pathological process of periodontitis, which provides clues to studying the pathogenesis of this disease and makes early diagnosis possible. Therefore, specific biomarkers associated with ERS are urgently needed to assist in the diagnosis of periodontitis to compensate for the drawbacks of the existing diagnostic modalities.

The Gene Expression Omnibus (GEO) is a public functional genomics database that contains high-throughput gene expression data submitted by research institutions worldwide [16]. Much of the data in GEO is only briefly analyzed and underutilized, so in-depth data analysis has become a common approach in bioinformatics research [17]. Machine learning is an emerging field in medicine, where computers analyze existing data, identify trends and patterns, and then predict the output values [18]. As an artificial intelligence technology, machine learning has broad applications in the medical field and can show powerful information mining and data computing capabilities when combined with bioinformatics frameworks.

In the present study, we obtained three periodontitis-related microarray datasets from the GEO database and randomized all samples into two sets. After identifying differentially expressed genes (DEGs) associated with ERS in the training set, machine learning algorithms were used to further screen out key DEGs, which were later verified in the validation set to identify potential biomarkers of periodontitis. We also analyzed immune cell infiltration in periodontitis tissues and explored the correlation between these potential biomarkers and immune cells.

## 2. Materials and Methods

### 2.1. Data Acquisition and Process

Three datasets of periodontitis (GSE10334, GES16134, and GES23586) were downloaded from the GEO database (https://www.ncbi.nlm.nih.gov/geo/). All three microarray datasets were based on the GPL570 platform. Next probe IDs were converted to gene symbols according to the annotation information of the platform. For the data in which a similar gene corresponded to multiple probes, gene expression was expressed as the average of multiple probes [19].

The three datasets were then merged and implemented with a batch correction to eliminate batch effects by using the “limma” and “sva” packages in the R software (version 4.1.1) [20]. Before further analysis, a total of 427 diseased and 136 healthy samples were randomly assigned in a 2 : 1 ratio using the R software, respectively. Two-thirds of the diseased samples and two-thirds of the healthy samples were randomly selected into the training set for subsequent screening of DEGs. The remaining diseased and healthy samples were assigned to the validation set.

ERS-related genes were acquired from the GeneCards database (https://www.genecards.org), and genes with relevance scores ≥ 10 were extracted for this study [21].

### 2.2. Identification of DEGs

ERS-related genes and their expression values in each sample were extracted from the expression profile of the training set. Differential expression analysis was performed using the “limma” package in the R software, and the selected conditions for DEGs were |log2FoldChange|>0.5 and adjusted *p* value < 0.05. Moreover, the “pheatmap” package and the “ggplot2” package were used to create the “heat plot” and “volcano plot” of the DEGs.

### 2.3. Functional Enrichment Analysis of DEGs

Functional enrichment analysis of all DEGs was performed with the “clusterProfiler” package in the R software [22]. Gene Ontology (GO) enriched the functional genes in different biological processes, cellular components, and molecular functions. Kyoto Encyclopedia of Genes and Genomes (KEGG) and Disease Ontology (DO) were enriched by gene pathways and diseases, respectively, to achieve effective clustering of DEGs.

### 2.4. Identification of Key DEGs Using Machine Learning and Construction of PPI Network

LASSO is a machine learning algorithm based on linear regression, which can assist researchers in improving prediction accuracy by screening gene expression data and is now widely used in bioinformatics [23]. Support vector machine-recursive feature elimination (SVM-RFE) is another machine learning algorithm that can iteratively filter out the feature subset with the highest accuracy rate for a large amount of data and can thus be used to identify potential biomarkers for diseases [24]. In this study, DEGs were further filtered by the LASSO algorithm from the “glmnet” package in the R software for 10-fold cross-validation. Meanwhile, the SVM-RFE algorithm from the “e1071” package has also been used; specifically, the size was set to 2 to 40 with a step size of 3, the rfeControl was set to functions with “caretFuncs” and method with “cv”, and the methods were set to “svmRadial.” To further improve the prediction accuracy and minimize the error rate, the Venn plot was used to obtain the overlapping genes of these two algorithms for subsequent analysis, which were identified as key DEGs.

Protein-protein interaction (PPI) networks were constructed using the STRING online platform (https://www.string-db.org) for the ERS-related key DEGs [25]. The minimum required interaction score was set at 0.4 of medium confidence, and the strength of data support was indicated by line thickness.

### 2.5. Verification of the Key DEGs' Diagnostic Value

Key DEGs were verified using samples from the validation set. Box plots were created using the “ggplot2” and “ggpubr” packages in the R software to show the expression of key DEGs in periodontitis and healthy tissues. The receiver operating characteristic (ROC) curves were plotted using the “pROC” package, and the area under the curve (AUC) was used to indicate the diagnostic value of genes [26]. In the present study, a gene was considered to have high predictive diagnostic efficiency and could be identified as a potential biomarker if its AUC was greater than 0.85 in both the training and validation sets.

### 2.6. Evaluation of Immune Cell Infiltration

Twenty-two immune cell subsets were evaluated for infiltration in periodontitis tissue using the CIBERSORT algorithm [27]. The “ggplot2” and “pheatmap” packages in the R software were used to visualize the relative content of these immune cells from all samples. The “corrplot” package was used to create the correlation heat map of individual immune cell subsets, and the “vioplot” package was used to create a violin plot, which showed the differences in immune cell infiltration between periodontitis and healthy periodontal tissues. Finally, we calculated the relationship between potential biomarkers and immune cell infiltration using Spearman correlation analysis and generated the visualization results using the “ggplot2” and “ggpubr” packages.

## 3. Results

**Figure 1:**
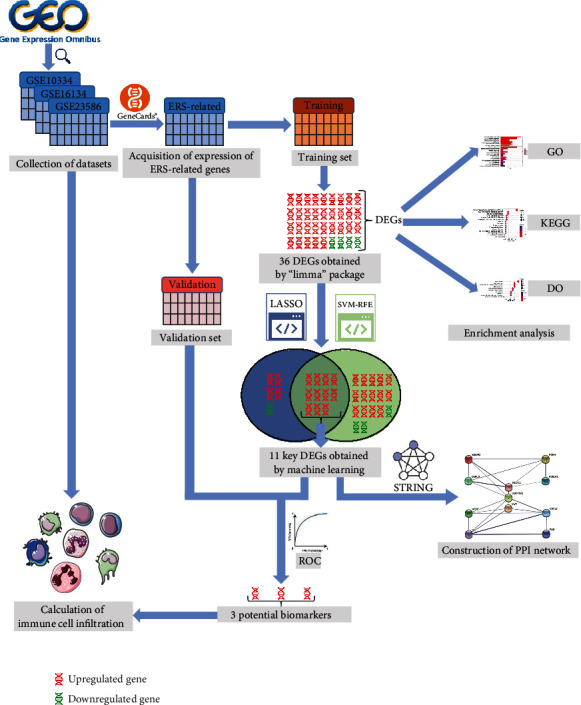
The overall process of the study.

The overall process of the study is shown in Figure 1.

### 3.1. Identification of DEGs of Periodontitis

**Figure 2:**
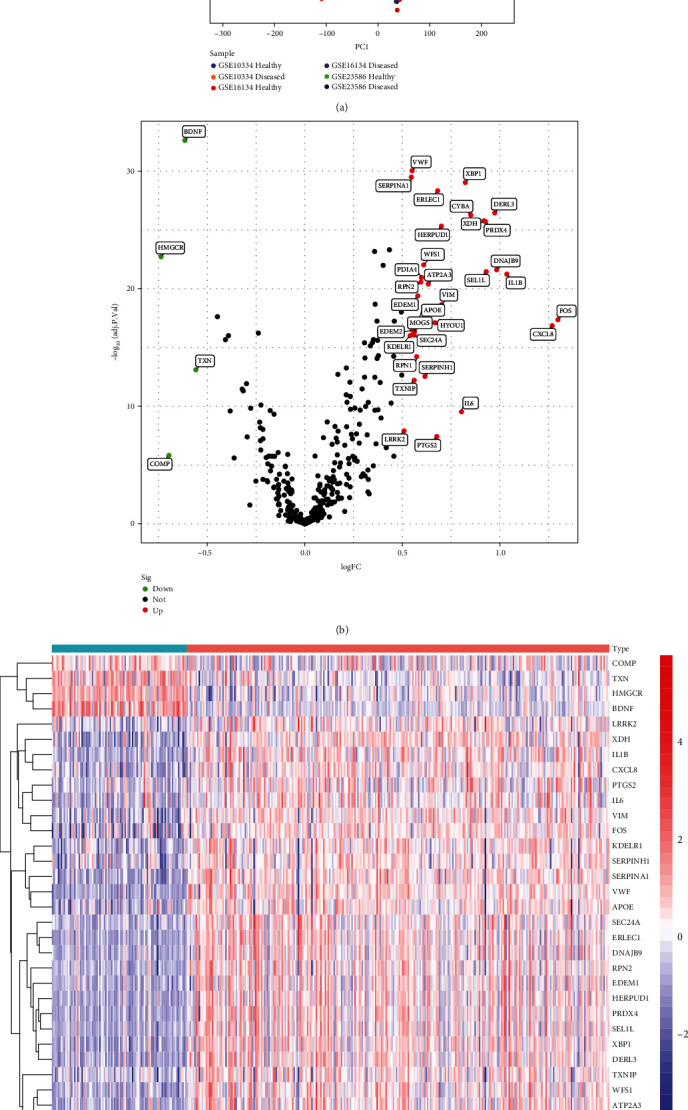
Identification of DEGs of periodontitis. (a) PCA plot of diseased and healthy periodontal tissue samples after the batch effect between GSE10334, GES16134, and GES1613 was removed. (b) Volcano plot of DEGs in training the set; green represented downregulated DEGs, black represented genes with no significant difference, and red represented upregulated DEGs. (c) Heat map of 36 DEGs with significant differences in expression between diseased and healthy periodontal samples.

Three microarray datasets for periodontitis were downloaded from the GEO database; the detailed characteristics of these datasets are shown in Table 1. The GSE10334, GES16134, and GES23585 datasets were merged and implemented with batch correction, and the principal component analysis (PCA) plot indicated that batch effects between samples had been removed after correction (Figure 2(a)). Next, all diseased and healthy samples were randomly portioned into the training and validation sets with a 2 : 1 ratio. The training set comprised 285 diseased and 91 healthy tissue samples, and the validation set comprised 142 diseased and 45 healthy tissue samples. The exact sample assignments are shown in Supplementary Materials: Table S1.

We obtained a total of 376 ERS-related genes with relevance scores ≥ 10 from the GeneCards database (Supplementary Materials: Table S2), 354 of which were present in the training set and used for differential expression analysis. A total of 36 DEGs were identified to meet the selection criteria of |log2FoldChange| > 0.5 and adjusted *p* value < 0.05. The 32 genes significantly upregulated in diseased tissues were indicated in red in the volcano plot, and the 4 markedly downregulated genes were indicated in green (Figure 2(b)). The heat map showed the global expression variation of the 36 DEGs in the samples (Figure 2(c)). The DEGs and their log2foldchange, average expression, *p* values, and adjusted *p* values are shown in Supplementary Materials: Table S3.

### 3.2. Functional Enrichment Analysis

**Figure 3:**
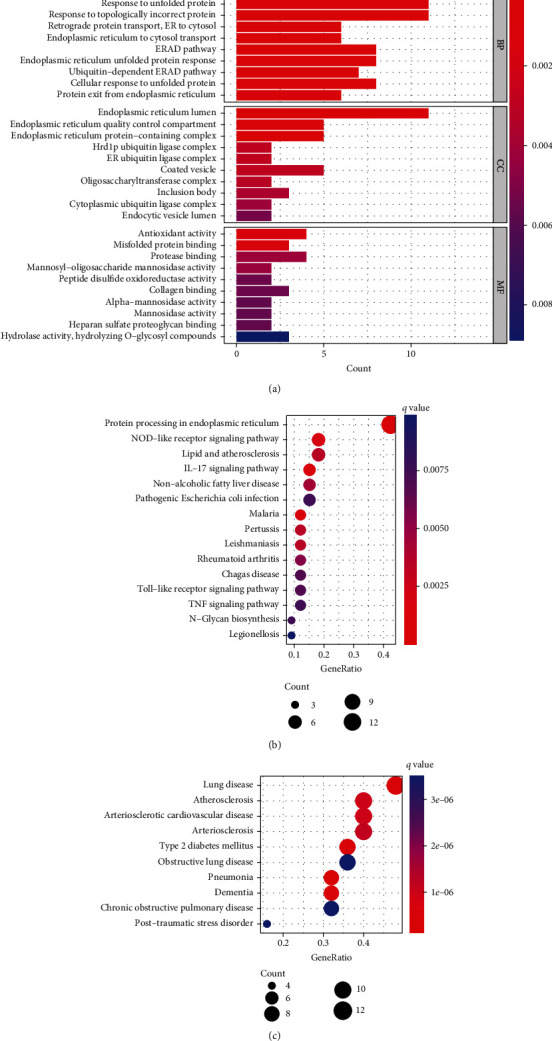
Functional enrichment analysis of the 36 DEGs. (a) Histogram of GO analysis for DEGs, including molecular processes, cellular components, and molecular functions. (b) Bubble diagram of KEGG analysis for DEGs. (c) Bubble diagram of DO analysis for DEGs.

To explore the biological processes and potential functions involved in 36 periodontitis DEGs within and outside the cell, we performed GO, KEGG, and DO enrichment analyses. The biological processes were mainly enriched in response to ERS, UPR, topologically incorrect protein, retrograde protein transport, ER to cytosol, and ER to cytosol transport. Enriched cellular components were also closely related to ERS, including ER lumen, ER quality control compartment, and ER protein-containing complex. The molecular functions were significantly correlated with antioxidant activity, misfolded protein binding, and protease binding (Figure 3(a)). The KEGG analysis showed that protein processing in ER, NOD-like receptor signaling pathway, lipid and atherosclerosis, and IL-17 signaling pathway were the most enriched (Figure 3(b)). Moreover, these DEGs were linked to lung disease, atherosclerosis, arteriosclerotic cardiovascular disease, and arteriosclerosis, according to the DO analysis (Figure 3(c)).

### 3.3. Identification of Key DEGs by Machine Learning

**Figure 4:**
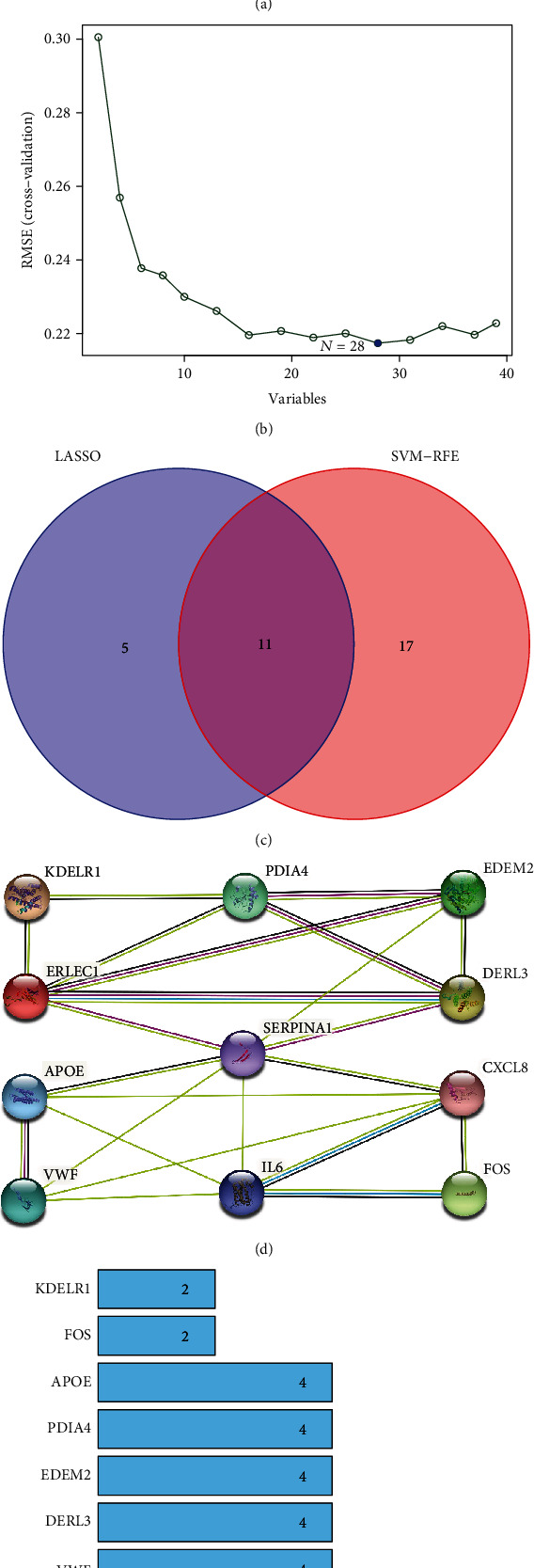
Machine learning and construction of PPI network. (a) Further screening of DEGs by LASSO regression algorithm. (b) Further screening of DEGs by SVM-RFE algorithm. (c) Venn plot of intersection genes between LASSO regression algorithm and SVM-RFE algorithm. (d) PPI network of 11 key DEGs of periodontitis. (e) Bar plot of 11 key genes' node degree in the PPI network.

In order to select key DEGs valuable for periodontitis diagnosis, we used two machine learning methods, LASSO and SVM-RFE, to further filter the above obtained DEGs. The LASSO regression screened 16 genes (Figure 4(a)), and the SVM-RFE algorithm yielded 28 outputs (Figure 4(b)). The 11 upregulated genes obtained by intersecting the results of the two methods were identified as key DEGs, including SERPINA1, ERLEC1, VWF, DERL3, PDIA4, FOS, CXCL8, EDEM2, APOE, KDELR1, and IL6 (Figure 4(c)).

In addition, we constructed the PPI network between proteins encoded by the 11 key DEGs in the STRING database to explore their interactions. The PPI network consisted of 23 edges and 11 nodes (Figure 4(d)), and the node degree of each protein had an average of 4.18 (Figure 4(e)). Moreover, the PPI enrichment *p* value was 1.58*e*-10.

### 3.4. Validation of Key DEGs and Screening of Potential Markers

To verify the generalizability of the key DEGs, we separately analyzed the ERS-related DEGs in GSE10334 and GSE16134. The GSE23586 was not worth analyzing as a separate dataset for its relatively small sample size. The results showed 29 and 39 ERS-related DEGs in GSE10334 and GSE16134, respectively. Moreover, we found that 10 of the 11 key DEGs identified by machine learning mentioned above were significantly upregulated in both major datasets, while KDELR1 was only upregulated in GSE16134 (Supplementary Materials: Figure S1).

**Figure 5:**
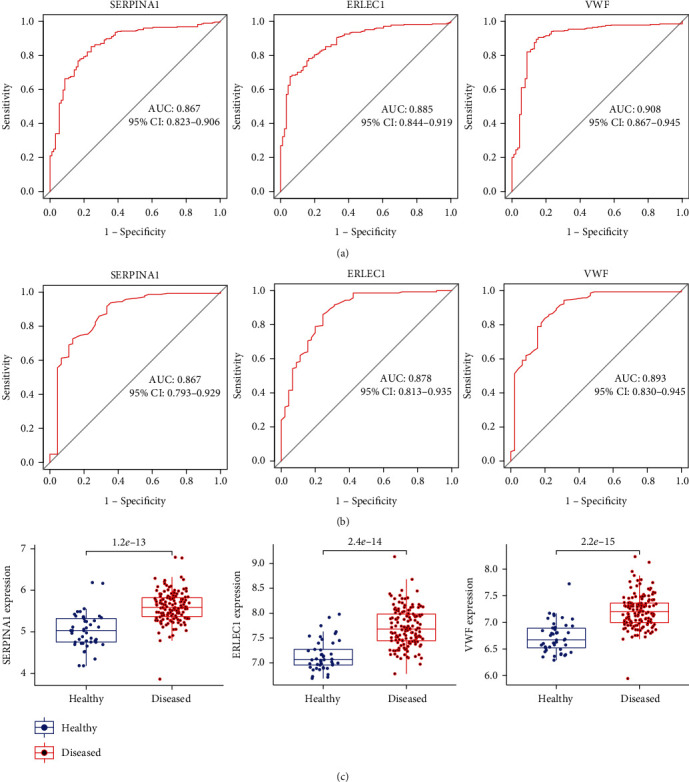
Evaluation of the diagnostic efficacy of three potential biomarkers of periodontitis. (a) ROC curves of SERPINA1, ERLEC1, and VWF in the training set. (b) ROC curves of SERPINA1, ERLEC1, and VWF in the validation set. (c) Box plots of differential expression levels of SERPINA1, ERLEC1, and VWF in diseased and healthy periodontal samples of the validation set.

We then plotted the ROC curves of the above key DEGs separately in the training and validation sets to further examine their diagnostic efficacy. Genes with AUC greater than 0.85 in both datasets were considered potential biomarkers. SERPINA1 (AUC: 0.867, 95% CI: 0.823-0.906), ERLEC1 (AUC: 0.885, 95% CI: 0.844-0.919), and VWF (AUC: 0.908, 95% CI: 0.867-0.945) showed a preferable diagnostic value in the training set (Figure 5(a)). After further confirmation by the validation set, the expression of these three genes was significantly higher in the diseased samples than in the healthy ones (Figure 5(c)), and the ROC curves of SERPINA1 (AUC: 0.867, 95% CI: 0.793-0.929), ERLEC1 (AUC: 0.878, 95% CI: 0.813-0.935), and VWF (AUC: 0.893, 95% CI: 0.830-0.945) also displayed a high diagnostic value (Figure 5(b)). Therefore, SERPINA1, ERLEC1, and VWF were selected as potential biomarkers of periodontitis. The ROC curves of the other key DEGs are shown in Supplementary Materials: Figure S2.

### 3.5. Immune Cell Infiltration Analysis

**Figure 6:**
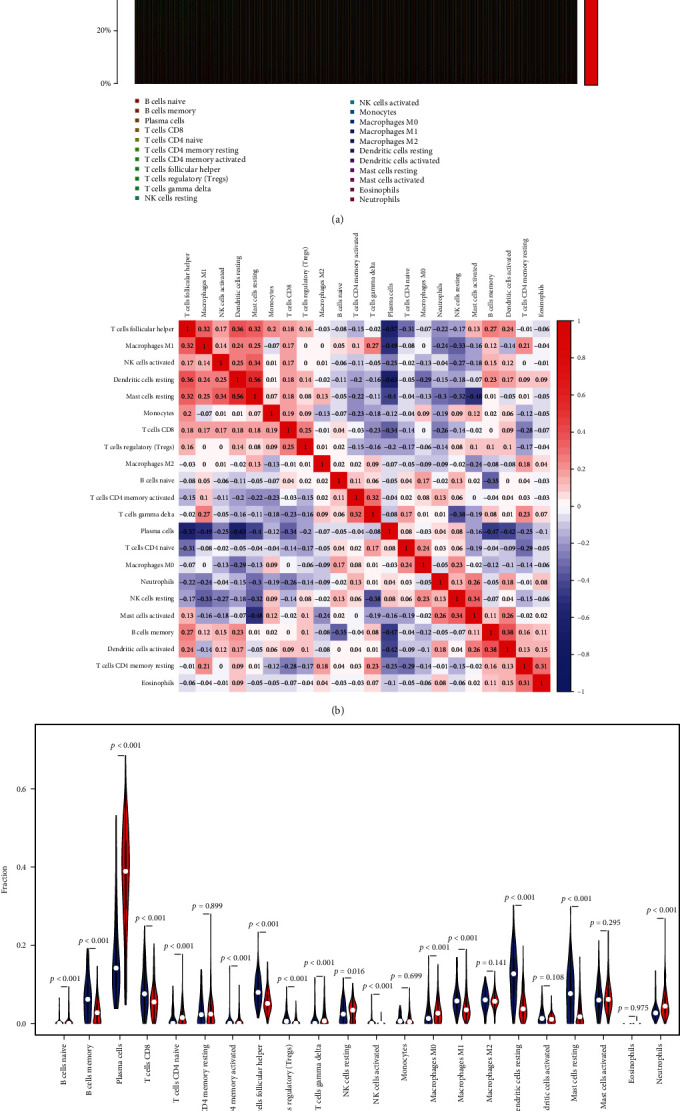
Calculation of immune cell infiltration in periodontitis by the CIBERSORT algorithm. (a) Bar plot of the relative proportions of immune cells. (b) Heat map of correlations between 22 immune cell subsets; red indicated a positive correlation between two cell subsets, white indicated no correlation between two cell subsets, and blue indicated a negative correlation between two cell subsets. (c) Violin plot of immune cell differences between diseased and healthy periodontal samples.

As an inflammatory disease, periodontitis has a host immune response that promotes tissue destruction, which may also involve ERS [14, 28]. To further investigate the role played by immune cells in periodontitis and the correlation between ERS-related genes and immune cells, we calculated immune cell infiltration using the CIBERSORT algorithm and the Spearman method separately. The relative proportions of immune cells in 427 diseased and 136 healthy periodontal tissue samples from GSE10334, GES16134, and GES1613 are shown in Figure 6(a). According to the correlation analysis among 22 immune cell subsets, resting dendritic cells and resting mast cells had the strongest positive correlation (*r* = 0.56), while plasma cells and resting dendritic cells had the strongest negative correlation (*r* = −0.63) (Figure 6(b)). Additionally, compared with healthy samples, periodontitis samples contained significantly lower resting dendritic cells, resting mast cells, follicular helper T cells, memory B cells, and M1 macrophages, while plasma cells and neutrophils were significantly higher (Figure 6(c)).

**Figure 7:**
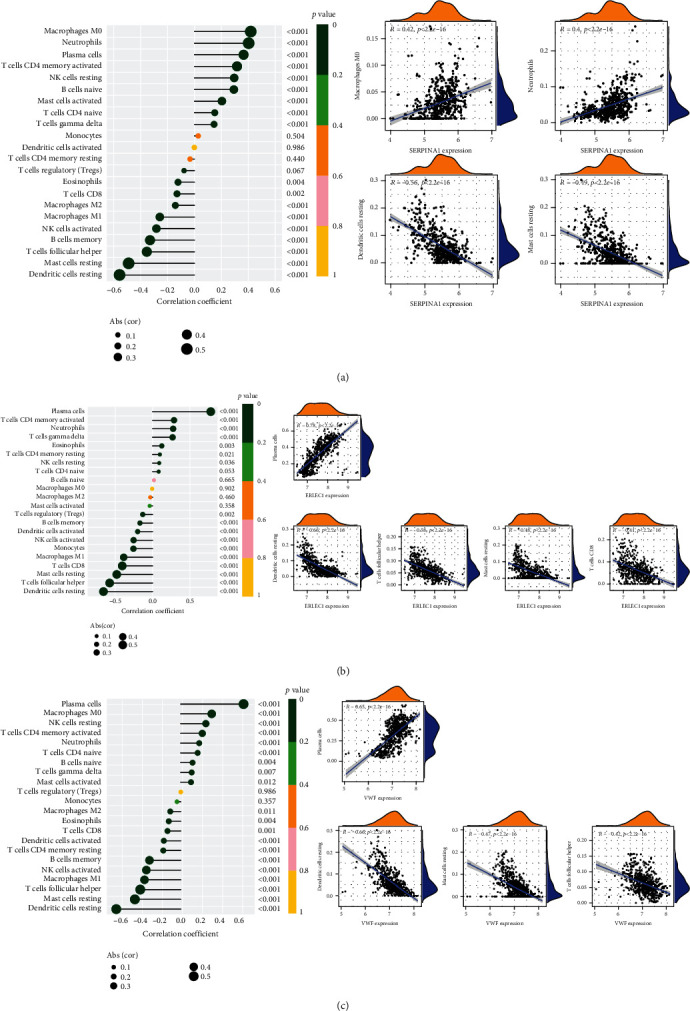
Correlation analysis between potential biomarkers of periodontitis and immune cells. (a) Lollipop plot and correlation plots of the correlations between SERPINA1 and immune cells. (b) Lollipop plot and correlation plots of the correlations between ERLEC1 and immune cells. (c) Lollipop plot and correlation plots of the correlations between VWF and immune cells. The correlation plots were displayed if |r| > 0.4 and *p* < 0.05.

Correlation analysis between the potential biomarkers and immune cells showed that the expression of SERPINA1 was positively correlated with M0 macrophages (*r* = 0.42, *p* = 2.8*e* − 25) and neutrophils (*r* = 0.4, *p* = 2.1*e* − 23) and negatively correlated with resting dendritic cells (*r* = −0.56, *p* = 4*e* − 47) and resting mast cells (*r* = −0.49, *p* = 3.3*e* − 35) (Figure 7(a)). The expression of ERLEC1 was positively correlated with plasma cells (*r* = 0.78, *p* = 3.5*e* − 116) and negatively correlated with resting dendritic cells (*r* = −0.66, *p* = 3.1*e* − 71), follicular helper T cells (*r* = −0.58, *p* = 5.8*e* − 51), resting mast cells (*r* = −0.48, *p* = 9.2*e* − 34), and CD8+ T cells (*r* = −0.41, *p* = 8.2*e* − 24) (Figure 7(b)). Similarly, the expression of VWF was positively correlated with plasma cells (*r* = 0.65, *p* = 5.3*e* − 68) and negatively correlated with resting dendritic cells (*r* = −0.66, *p* = 1.2*e* − 72), resting mast cells (*r* = −0.47, *p* = 1.5*e* − 32), and follicular helper T cells (*r* = −0.42, *p* = 5*e* − 25) (Figure 7(c)).

## 4. Discussion

Periodontitis is an inflammatory disease caused by the interactions between oral microorganisms and the host, but its pathogenesis is complex and still not fully investigated [29]. Currently, the diagnostic criteria for periodontitis are clinical parameters, which are not timely and accurate enough. Due to the lack of early diagnostic biomolecules, periodontal therapy is often conducted when periodontitis is already severe, which means a poor prognosis. Using bioinformatics methods to identify biomarkers can contribute to the early and accurate diagnosis of oral diseases and reduce the risk of disease progression, thus improving prognosis. However, there are no generally accepted specific biomarkers of periodontitis at present. ER is the main site of cellular protein folding, and the disruption of ER homeostasis can trigger ERS when misfolded proteins accumulate excessively, or calcium levels are altered too much. If the stress lasts too long or is too severe, it can cause irreversible damage to the cells and even induce cell death [30]. The association between ERS and periodontitis is being increasingly revealed, in which the activation of ERS can exacerbate periodontitis [31, 32]. Therefore, searching for ERS-related biomarkers and revealing their correlations with periodontitis could provide an important parameter for early diagnosis.

To our knowledge, this was the first study to identify ERS-related biomarkers of periodontitis based on microarray datasets. Single-cell sequencing and RNA-seq data that do not rely on predesigned probes are now increasingly used because of their high sensitivity and ability to detect novel genes [33, 34]. However, the samples of periodontitis using these two methods are now too few in the public platform to be analyzed on a large scale. Based on the clinical indexes provided in three datasets, CAL, PPD, and BOP, all patients included in the study met the criteria for periodontitis. Moreover, all sample data came from the same sequencing platform, all patients were nonsmokers and had no systemic diseases, and more than 98% were from the same country, which avoided interference from these confounding factors. The enrichment analysis results confirmed the strong correlation between DEGs and ERS-related biological functions, which further verified the involvement of ERS in periodontitis. The GO enrichment analysis of DEGs revealed that these genes are mainly associated with ERS-related biological processes and cellular components. Consistently, we obtained similar results in the KEGG pathway analysis, further probing that these DEGs are involved in protein processing in the ER. Moreover, our enrichment results are consistent with existing experimental studies, demonstrating the involvement of ER in the pathological pathway of periodontitis and proving the correlation between ERS and periodontitis [35].

To ensure that the key DEGs have a reliable prediction ability, we set a stringent selection criterion for the intersection between the two machine learning methods, LASSO and SVM-RFE. Because different algorithms produce different computing results, it is difficult to choose only one of the methods to achieve reliable findings. The STRING-based PPI network revealed significant interactions among key DEGs. In fact, cellular functions are completed by multiple proteins, rather than by individual proteins. SERPINA1, ERLEC1, and VWF, as crosstalk nodes, appear to be closely associated with other key genes and play an important core role in the whole biological molecular network. After further screening by the validation set, SERPINA1, ERLEC1, and VWF were selected as potential biomarkers of periodontitis.

SERPINA1 is the protein-encoding gene of alpha-1 antitrypsin (AAT). AAT is a serine protease inhibitor that inhibits neutrophil elastase, trypsin, and chymotrypsin by covalent binding [36]. A recent study found that SERPINA1 was a critical gene in breast cancer and periodontitis and was significantly associated with the prognosis of patients [37]. SERPINA1 also has anti-inflammatory effects in LPS-stimulated monocytes, which can enhance the release of the anti-inflammatory cytokine IL-10 and inhibit the synthesis and release of TNF-*α* and IL-1*β* in a concentration-dependent manner [38]. Besides, AAT has been found to have a potential role in mitigating bone loss [39]. In order to resist the tissue damage caused by tissue-destructive enzymes and reactive oxygen species (ROS) in periodontitis, a series of anti-inflammatory mediators were produced in response by cells [40]; this could explain the increase of SERPINA1 in the diseased samples of our study. In our study, SERPINA1 was the core gene in PPI and correlated with seven ERS-related genes including ERLEC1, VWF, EDEM2, DERL3, APOE, IL6, and CXCL8, suggesting that SERPINA1 may play an essential role in the pathological process of periodontitis.

ER lectin 1 (ERLEC1), also known as XTP3-B, is an ER-resident protein that selectively recognizes sugar moieties and targets improperly folded luminal proteins to the ER-associated degradation (ERAD) pathway, functioning as an ER quality control [41]. ERAD can protect cells from the adverse effects of ERS, but excessive ERAD can be harmful by disrupting cellular homeostasis and inducing apoptosis via UPR [42]. Moreover, when ERS induces excessive tissue apoptosis, destruction of periodontal soft tissues can occur [35]. A recent study has shown that proper expression levels of ERLEC1 are critical in osteogenic differentiation, and the occurrence of abnormal jaw development could be associated with pathogenic variants of ERLEC1 [43]. ERLEC1, the second highest scoring gene in the PPI network, was associated with five key DEGs, SERPINA1, EDEM2, DERL3, PDIA4, and KDELR1, showing that it may be a key linker molecule in the ERS functional module. However, there are still relatively few studies on ERLEC1, and no studies on the role of ERLEC1 in periodontitis have been reported so far. The correlation between them was presented for the first time in this paper, and future research is needed to confirm it.

Von Willebrand factor (VWF) is a multimeric glycoprotein synthesized in endothelial cells and megakaryocytes, which is mainly distributed in the plasma and plays a vital role in hemostasis by mediating platelet adhesion to subendothelial components after vascular injury [44]. There are intricate indirect and direct links between VWF and inflammation, and VWF can be massively released by endothelial cells to the extracellular milieu via cytokinesis as a response to inflammatory stimuli [45]. A study with 63 participants confirmed that VWF in peripheral blood was higher in patients with periodontitis than in controls [46]. It was also found that ERS in homocysteine-induced endothelial cells was accompanied by VWF deposition [47], which is consistent with our result. Meanwhile, the PPI network also revealed interrelationships between VWF and four ERS-related key genes, including SERPINA1, APOE, IL6, and CXCL8. However, VWF, as a marker of inflammation, remains to be investigated further in periodontitis.

Immune cells and immune responses are extensively involved in the progression of periodontitis; however, their specific roles and mechanisms are still unclear. An increasing number of articles have investigated the vital function of 22 immune cell subsets in periodontitis using the CIBERSORT algorithm. A study detected that the difference in immune cells between healthy periodontal tissues and periodontitis mainly included B cells, activated CD4+ memory T cells, resting dendritic cells, and neutrophils [48]. Another study revealed that the most upregulated immune cells in periodontitis tissues were neutrophils, and the most downregulated ones were Tregs [49]. In our study, we found that the infiltration of plasma cells and neutrophils was increased in periodontitis tissue samples while resting dendritic cells, resting mast cells, follicular helper T cells, memory B cells, and M1 macrophages were significantly reduced. However, the current evidence is still limited, and these results need to be supported by more studies.

According to the correlation analysis between SPRNINA1, ERLEC1, and VWF and immune cells, these three potential biomarkers of periodontitis were positively correlated with plasma cells and neutrophils and negatively correlated with resting dendritic cells, resting mast cells, follicular helper T cells, and CD8+ T cells. Interestingly, these three genes did not always show the same correlation with every immune cell subset; for example, SERPINA1 and VWF showed positive correlations with M0 macrophages, but ERLEC1 did not show a significant one with this type of cells. According to available studies, immune cell types are changed during the progression of periodontitis [50]. In the early lesion stage, neutrophils and lymphocytes are the major infiltrating cells. Neutrophils have an immunomodulatory function and are closely associated with the development of periodontitis. On the one hand, neutrophils act as the predominant anti-infection cells that play a protective role in clearing pathogens, and on the other hand, they can also produce ROS and proteases hydrolases that cause tissue damage and destruction [51–53]. At the same stage, lymphocyte subpopulations present in the gingival tissue are also involved in the immune response as important components of immune microenvironment [54]. And as the disease progresses, plasma cells become the main infiltrating cells in both established and advanced lesions. Plasma cells, the only antibody-producing cell type in the human body, account for approximately 50% of the cells in periodontitis lesions, with multiple functions and dominant roles in the host's immune response [55]. In addition, a recent study suggested that some IgG+ plasma cells could produce the anti-inflammatory cytokines IL-35 and IL-37 to regulate alveolar bone loss in periodontitis [56]. Furthermore, dendritic cells, as antigen-presenting cells that regulate the differentiation of T cells and induce destructive immunity, are also involved in the progression of periodontitis [57]. Although our findings on the correlations between SPRNINA1, ERLEC1, and VWF and immune infiltration suggested the possible involvement of these genes in immune cell regulation, the detailed mechanisms remain unclear and require further confirmation in future studies.

Our study revealed the role of ERS in periodontitis; in particular, we screened three important novel biomarkers associated with immune cell infiltration, which may contribute to the development of early diagnostic techniques for periodontitis and mitigate the risk of disease progression. In addition, by analyzing the immune microenvironment associated with biomarkers, we can tell the condition of periodontal tissues. And these findings may provide clues for precision medicine and prediction of treatment response in periodontitis.

However, some shortcomings need to be considered. First, our study was limited to the available data analysis, and cellular experiments, animal experiments, or clinical samples are needed for subsequent validation. Second, the datasets from public open-source databases lacked some clinicopathological features of periodontitis, such as specific clinical classification and follow-up information, preventing us from calculating the correlations between potential biomarkers and the occurrence, progression, and prognosis of the disease. Third, most of the data we used were from the same group in the USA, so the applicability of these results to populations in other regions is unknown. Lastly, further studies are required to determine the location of SERPINA1, ERLEC1, and VWF in ERS and investigate the mechanisms by which these three genes are linked to periodontitis.

## 5. Conclusions

Through machine learning, we identified three potential biomarkers of periodontitis, SERPINA1, ERLEC1, and VWF and found strong correlations between these ERS-related genes and immune cell infiltration. Our findings elucidated the role of ERS in periodontitis and provided a valuable basis for its accurate diagnosis.

## Figures and Tables

**Table 1:** Detailed information of the datasets used for analysis.

GEO accession ID	GSE10334	GES16134	GSE23586
Platform	GPL570	GPL570	GPL570
	Affymetrix Human Genome U133 Plus 2.0 Array	Affymetrix Human Genome U133 Plus 2.0 Array	Affymetrix Human Genome U133 Plus 2.0 Array
Participants	90	120	3
Systemically healthy	Yes	Yes	Yes
Smoking	No	No	No
Samples	247	310	6
Case (diseased)	183	241	3
	BOP (+), PPD ≥ 4 mm, and CAL ≥ 3 mm	BOP (+), PPD ≥ 4 mm, and CAL ≥ 3 mm	BOP (+), GI ≥ 1, PPD ≥ 5 mm, and CAL ≥ 5 mm
Control (healthy)	64	69	3
	BOP (-), PPD ≤ 4 mm, and CAL ≤ 2 mm	BOP (-), PPD ≤ 4 mm, and CAL ≤ 2 mm	No gingival inflammation, PPD ≤ 2 mm, and no CAL
Country	USA	USA	Japan
Authors	Demmer et al.	Demmer et al.	Kubota et al.

BOP: bleeding on probing; PPD: probing pocket depth; CAL: clinical attachment loss; GI: gingival index.

## Data Availability

The datasets used and/or analyzed during the current study are available from the corresponding authors on reasonable request.

## Supplementary Materials

Supplementary Materials Table S1: the exact sample assignments of the training set and validation set. Table S2: ERS-related genes obtained from GeneCards with relevance scores ≥10. Table S3: the DEGs and their differential expression characteristics. Figure S1: Venn plot of overlapping ERS-related DEGs between GSE10334, GSE16134, and machine learning. Figure S2: the ROC curves for other key DEGs.
