# Supplementary material for: Identification of Endoplasmic Reticulum Stress-Related Biomarkers of Periodontitis Based on Machine Learning: A Bioinformatics Analysis
Source: Dis Markers. 2022 Aug 29;2022:8611755. doi: 10.1155/2022/8611755 (PMC9444421; doi:10.1155/2022/8611755)
Supplement: Supplementary Materials — Table S1: the exact sample assignments of the training set and validation set. Table S2: ERS-related genes obtained from GeneCards with relevance scores ≥10. Table S3: the DEGs and their differential expression characteristics. Figure S1: Venn plot of overlapping ERS-related DEGs between GSE10334, GSE16134, and machine learning. Figure S2: the ROC curves for other key DEGs. [file 8611755.f1.zip › Table S1 (1).docx]

**Table S1:** The exact sample assignments of the training set and validation set.

| Sample | set | condition |
| --- | --- | --- |
| GSM261121 | training | diseased |
| GSM404191 | training | diseased |
| GSM404136 | training | diseased |
| GSM261216 | training | diseased |
| GSM404030 | training | diseased |
| GSM404185 | training | diseased |
| GSM404297 | training | diseased |
| GSM261255 | training | diseased |
| GSM404304 | training | diseased |
| GSM261110 | training | diseased |
| GSM261086 | training | diseased |
| GSM261104 | training | diseased |
| GSM404219 | training | diseased |
| GSM404208 | training | diseased |
| GSM261232 | training | diseased |
| GSM404207 | training | diseased |
| GSM261295 | training | diseased |
| GSM404237 | training | diseased |
| GSM404029 | training | diseased |
| GSM404307 | training | diseased |
| GSM261298 | training | diseased |
| GSM261297 | training | diseased |
| GSM404068 | training | diseased |
| GSM261313 | training | diseased |
| GSM261251 | training | diseased |
| GSM404065 | training | diseased |
| GSM404144 | training | diseased |
| GSM404018 | training | diseased |
| GSM404271 | training | diseased |
| GSM404086 | training | diseased |
| GSM404283 | training | diseased |
| GSM404083 | training | diseased |
| GSM261241 | training | diseased |
| GSM404076 | training | diseased |
| GSM404135 | training | diseased |
| GSM404276 | training | diseased |
| GSM404295 | training | diseased |
| GSM404299 | training | diseased |
| GSM404130 | training | diseased |
| GSM404027 | training | diseased |
| GSM404278 | training | diseased |
| GSM404193 | training | diseased |
| GSM261181 | training | diseased |
| GSM404249 | training | diseased |
| GSM261262 | training | diseased |
| GSM261278 | training | diseased |
| GSM261321 | training | diseased |
| GSM261192 | training | diseased |
| GSM261123 | training | diseased |
| GSM261128 | training | diseased |
| GSM261231 | training | diseased |
| GSM404302 | training | diseased |
| GSM404243 | training | diseased |
| GSM404033 | training | diseased |
| GSM261253 | training | diseased |
| GSM261172 | training | diseased |
| GSM404313 | training | diseased |
| GSM578532 | training | diseased |
| GSM261178 | training | diseased |
| GSM261271 | training | diseased |
| GSM404205 | training | diseased |
| GSM261150 | training | diseased |
| GSM404112 | training | diseased |
| GSM404164 | training | diseased |
| GSM404060 | training | diseased |
| GSM261214 | training | diseased |
| GSM261115 | training | diseased |
| GSM404070 | training | diseased |
| GSM261315 | training | diseased |
| GSM404223 | training | diseased |
| GSM261194 | training | diseased |
| GSM404290 | training | diseased |
| GSM404176 | training | diseased |
| GSM261264 | training | diseased |
| GSM404012 | training | diseased |
| GSM261279 | training | diseased |
| GSM404296 | training | diseased |
| GSM261092 | training | diseased |
| GSM261263 | training | diseased |
| GSM404170 | training | diseased |
| GSM404069 | training | diseased |
| GSM404286 | training | diseased |
| GSM404279 | training | diseased |
| GSM404272 | training | diseased |
| GSM261256 | training | diseased |
| GSM261119 | training | diseased |
| GSM404126 | training | diseased |
| GSM261287 | training | diseased |
| GSM404154 | training | diseased |
| GSM404055 | training | diseased |
| GSM261289 | training | diseased |
| GSM404066 | training | diseased |
| GSM404167 | training | diseased |
| GSM404150 | training | diseased |
| GSM261209 | training | diseased |
| GSM404011 | training | diseased |
| GSM404101 | training | diseased |
| GSM404047 | training | diseased |
| GSM261238 | training | diseased |
| GSM404090 | training | diseased |
| GSM404250 | training | diseased |
| GSM404161 | training | diseased |
| GSM404220 | training | diseased |
| GSM404224 | training | diseased |
| GSM404244 | training | diseased |
| GSM261284 | training | diseased |
| GSM404235 | training | diseased |
| GSM261261 | training | diseased |
| GSM404072 | training | diseased |
| GSM404305 | training | diseased |
| GSM404186 | training | diseased |
| GSM261252 | training | diseased |
| GSM404147 | training | diseased |
| GSM261273 | training | diseased |
| GSM404123 | training | diseased |
| GSM261163 | training | diseased |
| GSM261095 | training | diseased |
| GSM404020 | training | diseased |
| GSM261183 | training | diseased |
| GSM261189 | training | diseased |
| GSM261311 | training | diseased |
| GSM404174 | training | diseased |
| GSM404037 | training | diseased |
| GSM404098 | training | diseased |
| GSM261195 | training | diseased |
| GSM261230 | training | diseased |
| GSM404022 | training | diseased |
| GSM261144 | training | diseased |
| GSM404269 | training | diseased |
| GSM261190 | training | diseased |
| GSM404213 | training | diseased |
| GSM404121 | training | diseased |
| GSM404157 | training | diseased |
| GSM261153 | training | diseased |
| GSM404210 | training | diseased |
| GSM404048 | training | diseased |
| GSM404148 | training | diseased |
| GSM404052 | training | diseased |
| GSM404306 | training | diseased |
| GSM261126 | training | diseased |
| GSM261260 | training | diseased |
| GSM404140 | training | diseased |
| GSM404100 | training | diseased |
| GSM404258 | training | diseased |
| GSM404103 | training | diseased |
| GSM404159 | training | diseased |
| GSM261233 | training | diseased |
| GSM404257 | training | diseased |
| GSM404218 | training | diseased |
| GSM261292 | training | diseased |
| GSM404261 | training | diseased |
| GSM404097 | training | diseased |
| GSM261106 | training | diseased |
| GSM404151 | training | diseased |
| GSM261325 | training | diseased |
| GSM404077 | training | diseased |
| GSM404045 | training | diseased |
| GSM404089 | training | diseased |
| GSM404217 | training | diseased |
| GSM261212 | training | diseased |
| GSM404179 | training | diseased |
| GSM261282 | training | diseased |
| GSM261107 | training | diseased |
| GSM261322 | training | diseased |
| GSM261276 | training | diseased |
| GSM404247 | training | diseased |
| GSM404275 | training | diseased |
| GSM404044 | training | diseased |
| GSM404158 | training | diseased |
| GSM261299 | training | diseased |
| GSM261145 | training | diseased |
| GSM261175 | training | diseased |
| GSM404133 | training | diseased |
| GSM404038 | training | diseased |
| GSM261108 | training | diseased |
| GSM261198 | training | diseased |
| GSM261156 | training | diseased |
| GSM404063 | training | diseased |
| GSM404050 | training | diseased |
| GSM261275 | training | diseased |
| GSM404301 | training | diseased |
| GSM261093 | training | diseased |
| GSM261324 | training | diseased |
| GSM261281 | training | diseased |
| GSM261155 | training | diseased |
| GSM261215 | training | diseased |
| GSM404114 | training | diseased |
| GSM261265 | training | diseased |
| GSM261222 | training | diseased |
| GSM404129 | training | diseased |
| GSM404005 | training | diseased |
| GSM404113 | training | diseased |
| GSM404314 | training | diseased |
| GSM261136 | training | diseased |
| GSM404057 | training | diseased |
| GSM261129 | training | diseased |
| GSM261169 | training | diseased |
| GSM261173 | training | diseased |
| GSM261285 | training | diseased |
| GSM261141 | training | diseased |
| GSM404173 | training | diseased |
| GSM404202 | training | diseased |
| GSM404021 | training | diseased |
| GSM261160 | training | diseased |
| GSM404291 | training | diseased |
| GSM261244 | training | diseased |
| GSM261149 | training | diseased |
| GSM404092 | training | diseased |
| GSM404262 | training | diseased |
| GSM404084 | training | diseased |
| GSM261327 | training | diseased |
| GSM261133 | training | diseased |
| GSM404196 | training | diseased |
| GSM261242 | training | diseased |
| GSM261087 | training | diseased |
| GSM261267 | training | diseased |
| GSM261207 | training | diseased |
| GSM404288 | training | diseased |
| GSM261235 | training | diseased |
| GSM404310 | training | diseased |
| GSM261140 | training | diseased |
| GSM261125 | training | diseased |
| GSM261236 | training | diseased |
| GSM404153 | training | diseased |
| GSM404292 | training | diseased |
| GSM404259 | training | diseased |
| GSM404252 | training | diseased |
| GSM261167 | training | diseased |
| GSM404187 | training | diseased |
| GSM404040 | training | diseased |
| GSM404311 | training | diseased |
| GSM404234 | training | diseased |
| GSM261228 | training | diseased |
| GSM404232 | training | diseased |
| GSM261142 | training | diseased |
| GSM261305 | training | diseased |
| GSM404308 | training | diseased |
| GSM261101 | training | diseased |
| GSM261120 | training | diseased |
| GSM404282 | training | diseased |
| GSM261203 | training | diseased |
| GSM404266 | training | diseased |
| GSM404287 | training | diseased |
| GSM404119 | training | diseased |
| GSM261180 | training | diseased |
| GSM404199 | training | diseased |
| GSM404046 | training | diseased |
| GSM261248 | training | diseased |
| GSM261225 | training | diseased |
| GSM404041 | training | diseased |
| GSM404082 | training | diseased |
| GSM261187 | training | diseased |
| GSM404274 | training | diseased |
| GSM261227 | training | diseased |
| GSM404177 | training | diseased |
| GSM404277 | training | diseased |
| GSM404227 | training | diseased |
| GSM404078 | training | diseased |
| GSM404035 | training | diseased |
| GSM404201 | training | diseased |
| GSM261250 | training | diseased |
| GSM261200 | training | diseased |
| GSM404294 | training | diseased |
| GSM404094 | training | diseased |
| GSM261218 | training | diseased |
| GSM261182 | training | diseased |
| GSM261148 | training | diseased |
| GSM404015 | training | diseased |
| GSM404107 | training | diseased |
| GSM261130 | training | diseased |
| GSM404116 | training | diseased |
| GSM404238 | training | diseased |
| GSM404303 | training | diseased |
| GSM261300 | training | diseased |
| GSM404120 | training | diseased |
| GSM261259 | training | diseased |
| GSM261131 | training | diseased |
| GSM261114 | training | diseased |
| GSM261164 | training | diseased |
| GSM404265 | training | diseased |
| GSM404204 | training | diseased |
| GSM404221 | training | diseased |
| GSM404253 | training | diseased |
| GSM404145 | training | diseased |
| GSM404256 | training | diseased |
| GSM404032 | validation | diseased |
| GSM261310 | validation | diseased |
| GSM404192 | validation | diseased |
| GSM404293 | validation | diseased |
| GSM261270 | validation | diseased |
| GSM404127 | validation | diseased |
| GSM261094 | validation | diseased |
| GSM404142 | validation | diseased |
| GSM404226 | validation | diseased |
| GSM404298 | validation | diseased |
| GSM404241 | validation | diseased |
| GSM261170 | validation | diseased |
| GSM261185 | validation | diseased |
| GSM404255 | validation | diseased |
| GSM404254 | validation | diseased |
| GSM404111 | validation | diseased |
| GSM404034 | validation | diseased |
| GSM404106 | validation | diseased |
| GSM578530 | validation | diseased |
| GSM261147 | validation | diseased |
| GSM261245 | validation | diseased |
| GSM261221 | validation | diseased |
| GSM261111 | validation | diseased |
| GSM404198 | validation | diseased |
| GSM261132 | validation | diseased |
| GSM404209 | validation | diseased |
| GSM404263 | validation | diseased |
| GSM404009 | validation | diseased |
| GSM404268 | validation | diseased |
| GSM404231 | validation | diseased |
| GSM261103 | validation | diseased |
| GSM261208 | validation | diseased |
| GSM404118 | validation | diseased |
| GSM261269 | validation | diseased |
| GSM261301 | validation | diseased |
| GSM261100 | validation | diseased |
| GSM261302 | validation | diseased |
| GSM261210 | validation | diseased |
| GSM261176 | validation | diseased |
| GSM404309 | validation | diseased |
| GSM404285 | validation | diseased |
| GSM261331 | validation | diseased |
| GSM261191 | validation | diseased |
| GSM404091 | validation | diseased |
| GSM404181 | validation | diseased |
| GSM404087 | validation | diseased |
| GSM404137 | validation | diseased |
| GSM404056 | validation | diseased |
| GSM261204 | validation | diseased |
| GSM261304 | validation | diseased |
| GSM261247 | validation | diseased |
| GSM261197 | validation | diseased |
| GSM261157 | validation | diseased |
| GSM261239 | validation | diseased |
| GSM261201 | validation | diseased |
| GSM261211 | validation | diseased |
| GSM404182 | validation | diseased |
| GSM404300 | validation | diseased |
| GSM261161 | validation | diseased |
| GSM261224 | validation | diseased |
| GSM261312 | validation | diseased |
| GSM404156 | validation | diseased |
| GSM261330 | validation | diseased |
| GSM261213 | validation | diseased |
| GSM404043 | validation | diseased |
| GSM404194 | validation | diseased |
| GSM261316 | validation | diseased |
| GSM404211 | validation | diseased |
| GSM404079 | validation | diseased |
| GSM404214 | validation | diseased |
| GSM404104 | validation | diseased |
| GSM261118 | validation | diseased |
| GSM404081 | validation | diseased |
| GSM261135 | validation | diseased |
| GSM261124 | validation | diseased |
| GSM404141 | validation | diseased |
| GSM261166 | validation | diseased |
| GSM261186 | validation | diseased |
| GSM261258 | validation | diseased |
| GSM404124 | validation | diseased |
| GSM404168 | validation | diseased |
| GSM404312 | validation | diseased |
| GSM404281 | validation | diseased |
| GSM404110 | validation | diseased |
| GSM404058 | validation | diseased |
| GSM404289 | validation | diseased |
| GSM261098 | validation | diseased |
| GSM261219 | validation | diseased |
| GSM404073 | validation | diseased |
| GSM404138 | validation | diseased |
| GSM261319 | validation | diseased |
| GSM404165 | validation | diseased |
| GSM261288 | validation | diseased |
| GSM404230 | validation | diseased |
| GSM261105 | validation | diseased |
| GSM404014 | validation | diseased |
| GSM404188 | validation | diseased |
| GSM404008 | validation | diseased |
| GSM261152 | validation | diseased |
| GSM404109 | validation | diseased |
| GSM261328 | validation | diseased |
| GSM404212 | validation | diseased |
| GSM404062 | validation | diseased |
| GSM404280 | validation | diseased |
| GSM404162 | validation | diseased |
| GSM404184 | validation | diseased |
| GSM404095 | validation | diseased |
| GSM404026 | validation | diseased |
| GSM578534 | validation | diseased |
| GSM261139 | validation | diseased |
| GSM404189 | validation | diseased |
| GSM404240 | validation | diseased |
| GSM404051 | validation | diseased |
| GSM404019 | validation | diseased |
| GSM404197 | validation | diseased |
| GSM404006 | validation | diseased |
| GSM404067 | validation | diseased |
| GSM404024 | validation | diseased |
| GSM261294 | validation | diseased |
| GSM261112 | validation | diseased |
| GSM404264 | validation | diseased |
| GSM261318 | validation | diseased |
| GSM404132 | validation | diseased |
| GSM261158 | validation | diseased |
| GSM404216 | validation | diseased |
| GSM404229 | validation | diseased |
| GSM261090 | validation | diseased |
| GSM261179 | validation | diseased |
| GSM404061 | validation | diseased |
| GSM261097 | validation | diseased |
| GSM404178 | validation | diseased |
| GSM261291 | validation | diseased |
| GSM261089 | validation | diseased |
| GSM261308 | validation | diseased |
| GSM404171 | validation | diseased |
| GSM404023 | validation | diseased |
| GSM404139 | validation | diseased |
| GSM261307 | validation | diseased |
| GSM404246 | validation | diseased |
| GSM261274 | validation | diseased |
| GSM261290 | validation | diseased |
| GSM261268 | validation | diseased |
| GSM404036 | training | healthy |
| GSM261283 | training | healthy |
| GSM261113 | training | healthy |
| GSM404125 | training | healthy |
| GSM404228 | training | healthy |
| GSM261127 | training | healthy |
| GSM261243 | training | healthy |
| GSM404016 | training | healthy |
| GSM261323 | training | healthy |
| GSM404172 | training | healthy |
| GSM578533 | training | healthy |
| GSM404260 | training | healthy |
| GSM261286 | training | healthy |
| GSM261223 | training | healthy |
| GSM404115 | training | healthy |
| GSM404131 | training | healthy |
| GSM261146 | training | healthy |
| GSM404007 | training | healthy |
| GSM261246 | training | healthy |
| GSM404080 | training | healthy |
| GSM404152 | training | healthy |
| GSM261206 | training | healthy |
| GSM261240 | training | healthy |
| GSM261099 | training | healthy |
| GSM261091 | training | healthy |
| GSM404270 | training | healthy |
| GSM578529 | training | healthy |
| GSM404160 | training | healthy |
| GSM404190 | training | healthy |
| GSM261314 | training | healthy |
| GSM261117 | training | healthy |
| GSM261220 | training | healthy |
| GSM261134 | training | healthy |
| GSM404248 | training | healthy |
| GSM261296 | training | healthy |
| GSM261234 | training | healthy |
| GSM261277 | training | healthy |
| GSM261137 | training | healthy |
| GSM404128 | training | healthy |
| GSM404105 | training | healthy |
| GSM261159 | training | healthy |
| GSM404273 | training | healthy |
| GSM404042 | training | healthy |
| GSM404245 | training | healthy |
| GSM404239 | training | healthy |
| GSM404166 | training | healthy |
| GSM261138 | training | healthy |
| GSM404233 | training | healthy |
| GSM404155 | training | healthy |
| GSM404013 | training | healthy |
| GSM261280 | training | healthy |
| GSM261122 | training | healthy |
| GSM404071 | training | healthy |
| GSM261143 | training | healthy |
| GSM261249 | training | healthy |
| GSM404049 | training | healthy |
| GSM404180 | training | healthy |
| GSM261184 | training | healthy |
| GSM261332 | training | healthy |
| GSM261177 | training | healthy |
| GSM404200 | training | healthy |
| GSM404236 | training | healthy |
| GSM404074 | training | healthy |
| GSM404085 | training | healthy |
| GSM261309 | training | healthy |
| GSM261229 | training | healthy |
| GSM261266 | training | healthy |
| GSM404096 | training | healthy |
| GSM404028 | training | healthy |
| GSM261096 | training | healthy |
| GSM404117 | training | healthy |
| GSM404267 | training | healthy |
| GSM261306 | training | healthy |
| GSM404169 | training | healthy |
| GSM261205 | training | healthy |
| GSM261193 | training | healthy |
| GSM404134 | training | healthy |
| GSM261116 | training | healthy |
| GSM261257 | training | healthy |
| GSM404108 | training | healthy |
| GSM404149 | training | healthy |
| GSM404183 | training | healthy |
| GSM404206 | training | healthy |
| GSM404215 | training | healthy |
| GSM404054 | training | healthy |
| GSM261226 | training | healthy |
| GSM261109 | training | healthy |
| GSM261272 | training | healthy |
| GSM404222 | training | healthy |
| GSM261162 | training | healthy |
| GSM404143 | training | healthy |
| GSM404053 | validation | healthy |
| GSM261165 | validation | healthy |
| GSM404225 | validation | healthy |
| GSM261202 | validation | healthy |
| GSM404088 | validation | healthy |
| GSM261171 | validation | healthy |
| GSM404284 | validation | healthy |
| GSM404242 | validation | healthy |
| GSM261326 | validation | healthy |
| GSM404163 | validation | healthy |
| GSM404025 | validation | healthy |
| GSM261293 | validation | healthy |
| GSM261237 | validation | healthy |
| GSM261102 | validation | healthy |
| GSM261154 | validation | healthy |
| GSM261174 | validation | healthy |
| GSM404251 | validation | healthy |
| GSM261188 | validation | healthy |
| GSM404102 | validation | healthy |
| GSM404017 | validation | healthy |
| GSM404122 | validation | healthy |
| GSM261317 | validation | healthy |
| GSM404064 | validation | healthy |
| GSM404059 | validation | healthy |
| GSM261254 | validation | healthy |
| GSM404203 | validation | healthy |
| GSM404031 | validation | healthy |
| GSM261329 | validation | healthy |
| GSM261151 | validation | healthy |
| GSM404093 | validation | healthy |
| GSM261217 | validation | healthy |
| GSM261196 | validation | healthy |
| GSM404010 | validation | healthy |
| GSM404099 | validation | healthy |
| GSM261320 | validation | healthy |
| GSM261168 | validation | healthy |
| GSM261088 | validation | healthy |
| GSM404075 | validation | healthy |
| GSM404146 | validation | healthy |
| GSM404195 | validation | healthy |
| GSM404039 | validation | healthy |
| GSM261199 | validation | healthy |
| GSM404175 | validation | healthy |
| GSM578531 | validation | healthy |
| GSM261303 | validation | healthy |
