# Supplementary material for: Identification of Endoplasmic Reticulum Stress-Related Biomarkers of Periodontitis Based on Machine Learning: A Bioinformatics Analysis
Source: Dis Markers. 2022 Aug 29;2022:8611755. doi: 10.1155/2022/8611755 (PMC9444421; doi:10.1155/2022/8611755)
Supplement: Supplementary Materials — Table S1: the exact sample assignments of the training set and validation set. Table S2: ERS-related genes obtained from GeneCards with relevance scores ≥10. Table S3: the DEGs and their differential expression characteristics. Figure S1: Venn plot of overlapping ERS-related DEGs between GSE10334, GSE16134, and machine learning. Figure S2: the ROC curves for other key DEGs. [file 8611755.f1.zip › Table S2 (1).docx]

**Table S2:** ERS-related genes obtained from GeneCards with relevance scores >= 10.

| Symbol | Description | GC id | Score |
| --- | --- | --- | --- |
| HSPA5 | Heat Shock Protein Family A (Hsp70) Member 5 | GC09M125234 | 66.73 |
| ERN1 | Endoplasmic Reticulum To Nucleus Signaling 1 | GC17M064039 | 63.45 |
| ATP2A2 | ATPase Sarcoplasmic/Endoplasmic Reticulum Ca2+ Transporting 2 | GC12P110280 | 52.6 |
| ATP2A1 | ATPase Sarcoplasmic/Endoplasmic Reticulum Ca2+ Transporting 1 | GC16P038448 | 50.47 |
| EIF2AK3 | Eukaryotic Translation Initiation Factor 2 Alpha Kinase 3 | GC02M088556 | 49.01 |
| SERP1 | Stress Associated Endoplasmic Reticulum Protein 1 | GC03M150541 | 47.23 |
| XBP1 | X-Box Binding Protein 1 | GC22M028794 | 46.62 |
| ATP2A3 | ATPase Sarcoplasmic/Endoplasmic Reticulum Ca2+ Transporting 3 | GC17M003923 | 45.44 |
| ERP29 | Endoplasmic Reticulum Protein 29 | GC12P112013 | 43.59 |
| SERP2 | Stress Associated Endoplasmic Reticulum Protein Family Member 2 | GC13P044373 | 42.68 |
| ERP44 | Endoplasmic Reticulum Protein 44 | GC09M099979 | 42.36 |
| KDELR1 | KDEL Endoplasmic Reticulum Protein Retention Receptor 1 | GC19M048382 | 41.88 |
| ERAP1 | Endoplasmic Reticulum Aminopeptidase 1 | GC05M096760 | 40.62 |
| ERO1A | Endoplasmic Reticulum Oxidoreductase 1 Alpha | GC14M052640 | 40.47 |
| KDELR2 | KDEL Endoplasmic Reticulum Protein Retention Receptor 2 | GC07M006447 | 40.47 |
| OS9 | OS9 Endoplasmic Reticulum Lectin | GC12P057693 | 40.36 |
| HERPUD1 | Homocysteine Inducible ER Protein With Ubiquitin Like Domain 1 | GC16P056932 | 40 |
| ERLEC1 | Endoplasmic Reticulum Lectin 1 | GC02P053786 | 37.83 |
| VCP | Valosin Containing Protein | GC09M035056 | 37.82 |
| ERAP2 | Endoplasmic Reticulum Aminopeptidase 2 | GC05P096875 | 37.46 |
| ATF6 | Activating Transcription Factor 6 | GC01P161766 | 36.94 |
| KDELR3 | KDEL Endoplasmic Reticulum Protein Retention Receptor 3 | GC22P038468 | 36.78 |
| ERN2 | Endoplasmic Reticulum To Nucleus Signaling 2 | GC16M023690 | 35.83 |
| SEC16A | SEC16 Homolog A, Endoplasmic Reticulum Export Factor | GC09M136440 | 35.63 |
| DDIT3 | DNA Damage Inducible Transcript 3 | GC12M057516 | 35.04 |
| ERP27 | Endoplasmic Reticulum Protein 27 | GC12M014914 | 34.83 |
| ERO1B | Endoplasmic Reticulum Oxidoreductase 1 Beta | GC01M236216 | 34.63 |
| RER1 | Retention In Endoplasmic Reticulum Sorting Receptor 1 | GC01P002391 | 34.26 |
| CALR | Calreticulin | GC19P012938 | 32.26 |
| TP53 | Tumor Protein P53 | GC17M007661 | 32.19 |
| HSP90B1 | Heat Shock Protein 90 Beta Family Member 1 | GC12P103930 | 31.42 |
| CHERP | Calcium Homeostasis Endoplasmic Reticulum Protein | GC19M016517 | 31.36 |
| RYR2 | Ryanodine Receptor 2 | GC01P237042 | 31.34 |
| EIF2S1 | Eukaryotic Translation Initiation Factor 2 Subunit Alpha | GC14P067359 | 30.27 |
| ATF4 | Activating Transcription Factor 4 | GC22P039592 | 30.2 |
| ERMP1 | Endoplasmic Reticulum Metallopeptidase 1 | GC09M005749 | 29.62 |
| CANX | Calnexin | GC05P179678 | 29.11 |
| RYR1 | Ryanodine Receptor 1 | GC19P046621 | 28.16 |
| TRDN | Triadin | GC06M123198 | 27 |
| CPT2 | Carnitine Palmitoyltransferase 2 | GC01P053196 | 26.97 |
| SYVN1 | Synoviolin 1 | GC11M082240 | 26.19 |
| CASQ2 | Calsequestrin 2 | GC01M115700 | 25.98 |
| PDIA3 | Protein Disulfide Isomerase Family A Member 3 | GC15P043746 | 25.83 |
| NFE2L1 | NFE2 Like BZIP Transcription Factor 1 | GC17P049121 | 24.88 |
| MAPK8 | Mitogen-Activated Protein Kinase 8 | GC10P048306 | 24.55 |
| NFE2L2 | NFE2 Like BZIP Transcription Factor 2 | GC02M177227 | 23.69 |
| DERL1 | Derlin 1 | GC08M123013 | 23.67 |
| DERL2 | Derlin 2 | GC17M005471 | 23.66 |
| APP | Amyloid Beta Precursor Protein | GC21M025880 | 23.44 |
| DNAJC10 | DnaJ Heat Shock Protein Family (Hsp40) Member C10 | GC02P182716 | 22.84 |
| LMAN1 | Lectin, Mannose Binding 1 | GC18M059327 | 22.55 |
| TXNDC12 | Thioredoxin Domain Containing 12 | GC01M052020 | 22.42 |
| CALM1 | Calmodulin 1 | GC14P090396 | 22.34 |
| HMOX1 | Heme Oxygenase 1 | GC22P035380 | 22.28 |
| P4HB | Prolyl 4-Hydroxylase Subunit Beta | GC17M081843 | 22.1 |
| HYOU1 | Hypoxia Up-Regulated 1 | GC11M119139 | 22.05 |
| CASQ1 | Calsequestrin 1 | GC01P160190 | 22.02 |
| SOD1 | Superoxide Dismutase 1 | GC21P031659 | 21.94 |
| PSEN1 | Presenilin 1 | GC14P073136 | 21.88 |
| SREBF1 | Sterol Regulatory Element Binding Transcription Factor 1 | GC17M017810 | 21.72 |
| MAPK14 | Mitogen-Activated Protein Kinase 14 | GC06P073566 | 21.2 |
| SIL1 | SIL1 Nucleotide Exchange Factor | GC05M138957 | 21.05 |
| CASP3 | Caspase 3 | GC04M184627 | 20.95 |
| DNAJC3 | DnaJ Heat Shock Protein Family (Hsp40) Member C3 | GC13P095677 | 20.92 |
| CASP4 | Caspase 4 | GC11M104942 | 20.91 |
| BCL2 | BCL2 Apoptosis Regulator | GC18M063123 | 20.82 |
| DNAJB9 | DnaJ Heat Shock Protein Family (Hsp40) Member B9 | GC07P108569 | 20.82 |
| TNF | Tumor Necrosis Factor | GC06P073386 | 20.51 |
| PRKN | Parkin RBR E3 Ubiquitin Protein Ligase | GC06M161348 | 20.37 |
| AMFR | Autocrine Motility Factor Receptor | GC16M056361 | 20.37 |
| PPP1R15A | Protein Phosphatase 1 Regulatory Subunit 15A | GC19P048872 | 20.31 |
| MAP3K5 | Mitogen-Activated Protein Kinase Kinase Kinase 5 | GC06M136557 | 20.3 |
| BCAP31 | B Cell Receptor Associated Protein 31 | GC0XM153701 | 20.18 |
| CAT | Catalase | GC11P034460 | 20.12 |
| ITPR1 | Inositol 1,4,5-Trisphosphate Receptor Type 1 | GC03P004486 | 19.96 |
| PDIA4 | Protein Disulfide Isomerase Family A Member 4 | GC07M149003 | 19.73 |
| SLC6A4 | Solute Carrier Family 6 Member 4 | GC17M030194 | 19.56 |
| STIM1 | Stromal Interaction Molecule 1 | GC11P003855 | 19.48 |
| SEC23A | SEC23 Homolog A, COPII Coat Complex Component | GC14M039031 | 19.48 |
| CREB3 | CAMP Responsive Element Binding Protein 3 | GC09P038908 | 19.42 |
| H6PD | Hexose-6-Phosphate Dehydrogenase/Glucose 1-Dehydrogenase | GC01P009234 | 19.36 |
| DERL3 | Derlin 3 | GC22M023834 | 19.22 |
| G3BP1 | G3BP Stress Granule Assembly Factor 1 | GC05P151771 | 19.1 |
| BAX | BCL2 Associated X, Apoptosis Regulator | GC19P048954 | 19.08 |
| TXNDC5 | Thioredoxin Domain Containing 5 | GC06M007893 | 19.05 |
| CLU | Clusterin | GC08M027596 | 18.86 |
| JSRP1 | Junctional Sarcoplasmic Reticulum Protein 1 | GC19M002252 | 18.79 |
| STIP1 | Stress Induced Phosphoprotein 1 | GC11P064266 | 18.67 |
| INS | Insulin | GC11M002159 | 18.66 |
| MAPK1 | Mitogen-Activated Protein Kinase 1 | GC22M021759 | 18.45 |
| WFS1 | Wolframin ER Transmembrane Glycoprotein | GC04P006260 | 18.42 |
| MAN1B1 | Mannosidase Alpha Class 1B Member 1 | GC09P137086 | 18.31 |
| CFTR | CF Transmembrane Conductance Regulator | GC07P117287 | 18.28 |
| ATF6B | Activating Transcription Factor 6 Beta | GC06M032115 | 18.19 |
| LNPK | Lunapark, ER Junction Formation Factor | GC02M175924 | 18.08 |
| MANF | Mesencephalic Astrocyte Derived Neurotrophic Factor | GC03P051385 | 18.08 |
| SAR1B | Secretion Associated Ras Related GTPase 1B | GC05M134601 | 18.02 |
| OXSR1 | Oxidative Stress Responsive Kinase 1 | GC03P038183 | 17.87 |
| DNAJB11 | DnaJ Heat Shock Protein Family (Hsp40) Member B11 | GC03P186567 | 17.65 |
| RTN4 | Reticulon 4 | GC02M054934 | 17.48 |
| CRH | Corticotropin Releasing Hormone | GC08M066176 | 17.33 |
| HSPA4 | Heat Shock Protein Family A (Hsp70) Member 4 | GC05P133051 | 17.21 |
| STING1 | Stimulator Of Interferon Response CGAMP Interactor 1 | GC05M139476 | 17.13 |
| HSPA1A | Heat Shock Protein Family A (Hsp70) Member 1A | GC06P073408 | 17.01 |
| EPM2A | EPM2A Glucan Phosphatase, Laforin | GC06M145382 | 16.97 |
| CREB3L1 | CAMP Responsive Element Binding Protein 3 Like 1 | GC11P046353 | 16.91 |
| FOS | Fos Proto-Oncogene, AP-1 Transcription Factor Subunit | GC14P075278 | 16.82 |
| FKBP14 | FKBP Prolyl Isomerase 14 | GC07M030010 | 16.69 |
| PARK7 | Parkinsonism Associated Deglycase | GC01P008066 | 16.67 |
| SEL1L | SEL1L Adaptor Subunit Of ERAD E3 Ubiquitin Ligase | GC14M081471 | 16.5 |
| KCNH2 | Potassium Voltage-Gated Channel Subfamily H Member 2 | GC07M150944 | 16.46 |
| SELENOS | Selenoprotein S | GC15M113606 | 16.41 |
| KCNQ1 | Potassium Voltage-Gated Channel Subfamily Q Member 1 | GC11P002444 | 16.38 |
| MIA2 | MIA SH3 Domain ER Export Factor 2 | GC14P039230 | 16.25 |
| ATP13A1 | ATPase 13A1 | GC19M019645 | 16.23 |
| IL6 | Interleukin 6 | GC07P022725 | 16.23 |
| HSF1 | Heat Shock Transcription Factor 1 | GC08P144291 | 16.11 |
| SIRT1 | Sirtuin 1 | GC10P067884 | 16.1 |
| ABL1 | ABL Proto-Oncogene 1, Non-Receptor Tyrosine Kinase | GC09P130713 | 16.1 |
| DDRGK1 | DDRGK Domain Containing 1 | GC20M003253 | 16.08 |
| BDNF | Brain Derived Neurotrophic Factor | GC11M027654 | 15.62 |
| ERGIC3 | ERGIC And Golgi 3 | GC20P035542 | 15.59 |
| SEC61A1 | SEC61 Translocon Subunit Alpha 1 | GC03P128051 | 15.27 |
| G3BP2 | G3BP Stress Granule Assembly Factor 2 | GC04M075642 | 15.21 |
| HSPA8 | Heat Shock Protein Family A (Hsp70) Member 8 | GC11M123057 | 15.19 |
| PDIA2 | Protein Disulfide Isomerase Family A Member 2 | GC16P008759 | 15.19 |
| SIGMAR1 | Sigma Non-Opioid Intracellular Receptor 1 | GC09M034634 | 15.17 |
| SURF4 | Surfeit 4 | GC09M133361 | 15.16 |
| HSP90AA1 | Heat Shock Protein 90 Alpha Family Class A Member 1 | GC14M102080 | 15.16 |
| MBTPS2 | Membrane Bound Transcription Factor Peptidase, Site 2 | GC0XP021839 | 15.14 |
| LMNA | Lamin A/C | GC01P156082 | 15.11 |
| IL1B | Interleukin 1 Beta | GC02M112829 | 15.08 |
| BAG6 | BAG Cochaperone 6 | GC06M031639 | 15.03 |
| RPN1 | Ribophorin I | GC03M128619 | 14.98 |
| PTPN1 | Protein Tyrosine Phosphatase Non-Receptor Type 1 | GC20P050510 | 14.97 |
| PKP2 | Plakophilin 2 | GC12M032790 | 14.9 |
| HSPB1 | Heat Shock Protein Family B (Small) Member 1 | GC07P076302 | 14.89 |
| MTOR | Mechanistic Target Of Rapamycin Kinase | GC01M011106 | 14.85 |
| SELENON | Selenoprotein N | GC01P025800 | 14.82 |
| INSIG1 | Insulin Induced Gene 1 | GC07P155297 | 14.7 |
| ERLIN2 | ER Lipid Raft Associated 2 | GC08P037736 | 14.66 |
| CXCL8 | C-X-C Motif Chemokine Ligand 8 | GC04P073740 | 14.63 |
| NOS3 | Nitric Oxide Synthase 3 | GC07P150990 | 14.62 |
| GSR | Glutathione-Disulfide Reductase | GC08M030678 | 14.55 |
| CYCS | Cytochrome C, Somatic | GC07M025118 | 14.43 |
| NR3C1 | Nuclear Receptor Subfamily 3 Group C Member 1 | GC05M143277 | 14.4 |
| CREB3L2 | CAMP Responsive Element Binding Protein 3 Like 2 | GC07M137874 | 14.39 |
| SQSTM1 | Sequestosome 1 | GC05P179806 | 14.38 |
| SREBF2 | Sterol Regulatory Element Binding Transcription Factor 2 | GC22P041833 | 14.35 |
| VAPB | VAMP Associated Protein B And C | GC20P058389 | 14.29 |
| APOE | Apolipoprotein E | GC19P047219 | 14.27 |
| SELENOK | Selenoprotein K | GC03M053884 | 14.2 |
| ANK2 | Ankyrin 2 | GC04P112706 | 14.16 |
| NHLRC1 | NHL Repeat Containing E3 Ubiquitin Protein Ligase 1 | GC06M018120 | 14.09 |
| TGFB1 | Transforming Growth Factor Beta 1 | GC19M041301 | 14.08 |
| SCN5A | Sodium Voltage-Gated Channel Alpha Subunit 5 | GC03M038549 | 14.05 |
| UBC | Ubiquitin C | GC12M124911 | 13.98 |
| EIF2AK2 | Eukaryotic Translation Initiation Factor 2 Alpha Kinase 2 | GC02M037099 | 13.97 |
| JUN | Jun Proto-Oncogene, AP-1 Transcription Factor Subunit | GC01M058780 | 13.89 |
| SEC31A | SEC31 Homolog A, COPII Coat Complex Component | GC04M082818 | 13.88 |
| SOD2 | Superoxide Dismutase 2 | GC06M159669 | 13.85 |
| TMED4 | Transmembrane P24 Trafficking Protein 4 | GC07M044577 | 13.82 |
| ADIPOQ | Adiponectin, C1Q And Collagen Domain Containing | GC03P186842 | 13.79 |
| NOTCH3 | Notch Receptor 3 | GC19M015159 | 13.77 |
| SCAP | SREBF Chaperone | GC03M047413 | 13.75 |
| SEC13 | SEC13 Homolog, Nuclear Pore And COPII Coat Complex Component | GC03M010293 | 13.75 |
| EDEM1 | ER Degradation Enhancing Alpha-Mannosidase Like Protein 1 | GC03P005187 | 13.71 |
| MIA3 | MIA SH3 Domain ER Export Factor 3 | GC01P222618 | 13.71 |
| SEC24A | SEC24 Homolog A, COPII Coat Complex Component | GC05P134647 | 13.7 |
| RTN3 | Reticulon 3 | GC11P063700 | 13.68 |
| ESR1 | Estrogen Receptor 1 | GC06P151656 | 13.55 |
| PARP1 | Poly(ADP-Ribose) Polymerase 1 | GC01M226360 | 13.52 |
| SEC24B | SEC24 Homolog B, COPII Coat Complex Component | GC04P109433 | 13.51 |
| ASPH | Aspartate Beta-Hydroxylase | GC08M061500 | 13.5 |
| TRAM1 | Translocation Associated Membrane Protein 1 | GC08M070573 | 13.5 |
| LMAN2 | Lectin, Mannose Binding 2 | GC05M177710 | 13.5 |
| CKAP4 | Cytoskeleton Associated Protein 4 | GC12M106237 | 13.47 |
| CALM3 | Calmodulin 3 | GC19P046601 | 13.46 |
| CREB3L3 | CAMP Responsive Element Binding Protein 3 Like 3 | GC19P004153 | 13.46 |
| PSEN2 | Presenilin 2 | GC01P226870 | 13.43 |
| EIF2AK1 | Eukaryotic Translation Initiation Factor 2 Alpha Kinase 1 | GC07M006022 | 13.37 |
| PTGS2 | Prostaglandin-Endoperoxide Synthase 2 | GC01M186640 | 13.35 |
| BSCL2 | BSCL2 Lipid Droplet Biogenesis Associated, Seipin | GC11M082132 | 13.34 |
| SNCA | Synuclein Alpha | GC04M089724 | 13.33 |
| TXN | Thioredoxin | GC09M110243 | 13.32 |
| CD4 | CD4 Molecule | GC12P006786 | 13.3 |
| MAPK10 | Mitogen-Activated Protein Kinase 10 | GC04M085990 | 13.2 |
| ERLIN1 | ER Lipid Raft Associated 1 | GC10M100150 | 13.19 |
| ERGIC2 | ERGIC And Golgi 2 | GC12M029337 | 13.19 |
| RRBP1 | Ribosome Binding Protein 1 | GC20M017613 | 13.18 |
| HMGCR | 3-Hydroxy-3-Methylglutaryl-CoA Reductase | GC05P075336 | 13.17 |
| APOB | Apolipoprotein B | GC02M020956 | 13.16 |
| CASP9 | Caspase 9 | GC01M015491 | 13.14 |
| CRHR1 | Corticotropin Releasing Hormone Receptor 1 | GC17P045784 | 13.11 |
| VWF | Von Willebrand Factor | GC12M005917 | 13.02 |
| RAB1A | RAB1A, Member RAS Oncogene Family | GC02M065072 | 12.98 |
| ATL3 | Atlastin GTPase 3 | GC11M063624 | 12.94 |
| ARL6IP1 | ADP Ribosylation Factor Like GTPase 6 Interacting Protein 1 | GC16M019050 | 12.92 |
| TOR1A | Torsin Family 1 Member A | GC09M129812 | 12.92 |
| TAPBP | TAP Binding Protein | GC06M033299 | 12.9 |
| ATF3 | Activating Transcription Factor 3 | GC01P212565 | 12.86 |
| UFL1 | UFM1 Specific Ligase 1 | GC06P096521 | 12.81 |
| CRP | C-Reactive Protein | GC01M159724 | 12.8 |
| CASP8 | Caspase 8 | GC02P201233 | 12.8 |
| BNIP1 | BCL2 Interacting Protein 1 | GC05P173144 | 12.8 |
| RAB1B | RAB1B, Member RAS Oncogene Family | GC11P068554 | 12.79 |
| YIPF5 | Yip1 Domain Family Member 5 | GC05M144158 | 12.77 |
| MPO | Myeloperoxidase | GC17M058269 | 12.71 |
| STIM2 | Stromal Interaction Molecule 2 | GC04P026859 | 12.7 |
| AKT1 | AKT Serine/Threonine Kinase 1 | GC14M104769 | 12.65 |
| HSPA9 | Heat Shock Protein Family A (Hsp70) Member 9 | GC05M138554 | 12.63 |
| SESN2 | Sestrin 2 | GC01P028270 | 12.56 |
| LRRK2 | Leucine Rich Repeat Kinase 2 | GC12P040196 | 12.55 |
| PPIB | Peptidylprolyl Isomerase B | GC15M064155 | 12.5 |
| VEGFA | Vascular Endothelial Growth Factor A | GC06P043770 | 12.49 |
| INSIG2 | Insulin Induced Gene 2 | GC02P118088 | 12.48 |
| CAV3 | Caveolin 3 | GC03P008733 | 12.48 |
| TMED2 | Transmembrane P24 Trafficking Protein 2 | GC12P123584 | 12.46 |
| CISD2 | CDGSH Iron Sulfur Domain 2 | GC04P102868 | 12.44 |
| UGGT1 | UDP-Glucose Glycoprotein Glucosyltransferase 1 | GC02P128091 | 12.38 |
| P4HTM | Prolyl 4-Hydroxylase, Transmembrane | GC03P049484 | 12.3 |
| CALM2 | Calmodulin 2 | GC02M047124 | 12.26 |
| PLN | Phospholamban | GC06P118548 | 12.25 |
| ALG1 | ALG1 Chitobiosyldiphosphodolichol Beta-Mannosyltransferase | GC16P005033 | 12.22 |
| KEAP1 | Kelch Like ECH Associated Protein 1 | GC19M010486 | 12.21 |
| JPH2 | Junctophilin 2 | GC20M044111 | 12.21 |
| SEC61B | SEC61 Translocon Subunit Beta | GC09P099222 | 12.18 |
| CACNA1C | Calcium Voltage-Gated Channel Subunit Alpha1 C | GC12P001970 | 12.15 |
| SEC24C | SEC24 Homolog C, COPII Coat Complex Component | GC10P073744 | 12.14 |
| TAP1 | Transporter 1, ATP Binding Cassette Subfamily B Member | GC06M059128 | 12.09 |
| DMD | Dystrophin | GC0XM031097 | 12.08 |
| CCL2 | C-C Motif Chemokine Ligand 2 | GC17P034255 | 12.08 |
| SERPINA1 | Serpin Family A Member 1 | GC14M094376 | 12.06 |
| RSAD2 | Radical S-Adenosyl Methionine Domain Containing 2 | GC02P006865 | 12.04 |
| DDX3X | DEAD-Box Helicase 3 X-Linked | GC0XP041333 | 12.04 |
| SLN | Sarcolipin | GC11M107709 | 11.99 |
| HERPUD2 | HERPUD Family Member 2 | GC07M035632 | 11.97 |
| TMBIM6 | Transmembrane BAX Inhibitor Motif Containing 6 | GC12P049707 | 11.96 |
| PDIA6 | Protein Disulfide Isomerase Family A Member 6 | GC02M010784 | 11.88 |
| SEC62 | SEC62 Homolog, Preprotein Translocation Factor | GC03P169966 | 11.87 |
| STX17 | Syntaxin 17 | GC09P099906 | 11.87 |
| BCL2L1 | BCL2 Like 1 | GC20M031664 | 11.84 |
| GET3 | Guided Entry Of Tail-Anchored Proteins Factor 3, ATPase | GC19P012737 | 11.78 |
| RPS27A | Ribosomal Protein S27a | GC02P055231 | 11.77 |
| PREB | Prolactin Regulatory Element Binding | GC02M027130 | 11.76 |
| HLA-B | Major Histocompatibility Complex, Class I, B | GC06M059025 | 11.72 |
| COPB1 | COPI Coat Complex Subunit Beta 1 | GC11M014436 | 11.69 |
| DDOST | Dolichyl-Diphosphooligosaccharide--Protein Glycosyltransferase Non-Catalytic Subunit | GC01M020651 | 11.69 |
| SGK1 | Serum/Glucocorticoid Regulated Kinase 1 | GC06M134169 | 11.68 |
| SEC63 | SEC63 Homolog, Protein Translocation Regulator | GC06M107867 | 11.68 |
| ORAI1 | ORAI Calcium Release-Activated Calcium Modulator 1 | GC12P125118 | 11.67 |
| PRKCD | Protein Kinase C Delta | GC03P053156 | 11.65 |
| GBF1 | Golgi Brefeldin A Resistant Guanine Nucleotide Exchange Factor 1 | GC10P102245 | 11.65 |
| PRNP | Prion Protein | GC20P004686 | 11.64 |
| SLC37A4 | Solute Carrier Family 37 Member 4 | GC11M119024 | 11.63 |
| EIF4G1 | Eukaryotic Translation Initiation Factor 4 Gamma 1 | GC03P184314 | 11.6 |
| DNAH8 | Dynein Axonemal Heavy Chain 8 | GC06P073594 | 11.56 |
| PDCD6 | Programmed Cell Death 6 | GC05P000272 | 11.55 |
| IER3IP1 | Immediate Early Response 3 Interacting Protein 1 | GC18M047152 | 11.55 |
| EDEM2 | ER Degradation Enhancing Alpha-Mannosidase Like Protein 2 | GC20M035115 | 11.54 |
| BAK1 | BCL2 Antagonist/Killer 1 | GC06M033572 | 11.54 |
| ESYT1 | Extended Synaptotagmin 1 | GC12P056928 | 11.53 |
| UBE2J1 | Ubiquitin Conjugating Enzyme E2 J1 | GC06M089326 | 11.52 |
| SAR1A | Secretion Associated Ras Related GTPase 1A | GC10M070147 | 11.51 |
| ATL1 | Atlastin GTPase 1 | GC14P050532 | 11.51 |
| MYOC | Myocilin | GC01M171604 | 11.5 |
| GJB2 | Gap Junction Protein Beta 2 | GC13M020187 | 11.49 |
| CAPN3 | Calpain 3 | GC15P042359 | 11.44 |
| SERPINH1 | Serpin Family H Member 1 | GC11P075562 | 11.43 |
| XDH | Xanthine Dehydrogenase | GC02M031334 | 11.41 |
| MAPK9 | Mitogen-Activated Protein Kinase 9 | GC05M180244 | 11.41 |
| VIM | Vimentin | GC10P017227 | 11.4 |
| TRIM13 | Tripartite Motif Containing 13 | GC13P049995 | 11.4 |
| RNF139 | Ring Finger Protein 139 | GC08P124474 | 11.4 |
| HTRA2 | HtrA Serine Peptidase 2 | GC02P074529 | 11.37 |
| CANT1 | Calcium Activated Nucleotidase 1 | GC17M078992 | 11.36 |
| EIF2AK4 | Eukaryotic Translation Initiation Factor 2 Alpha Kinase 4 | GC15P039934 | 11.3 |
| OSBPL8 | Oxysterol Binding Protein Like 8 | GC12M076354 | 11.26 |
| TMED10 | Transmembrane P24 Trafficking Protein 10 | GC14M075132 | 11.24 |
| PINK1 | PTEN Induced Kinase 1 | GC01P020634 | 11.17 |
| TMEM33 | Transmembrane Protein 33 | GC04P041937 | 11.17 |
| TECRL | Trans-2,3-Enoyl-CoA Reductase Like | GC04M064275 | 11.16 |
| CAV1 | Caveolin 1 | GC07P116524 | 11.13 |
| SOAT1 | Sterol O-Acyltransferase 1 | GC01P179262 | 11.13 |
| EDEM3 | ER Degradation Enhancing Alpha-Mannosidase Like Protein 3 | GC01M184690 | 11.12 |
| TFG | Trafficking From ER To Golgi Regulator | GC03P100709 | 11.11 |
| TOR1B | Torsin Family 1 Member B | GC09P129803 | 11.09 |
| NOX4 | NADPH Oxidase 4 | GC11M089324 | 11.09 |
| HSD17B10 | Hydroxysteroid 17-Beta Dehydrogenase 10 | GC0XM053431 | 11.07 |
| RYR3 | Ryanodine Receptor 3 | GC15P033310 | 11.04 |
| VAPA | VAMP Associated Protein A | GC18P009904 | 11.04 |
| PRKAA1 | Protein Kinase AMP-Activated Catalytic Subunit Alpha 1 | GC05M040759 | 11 |
| RTN1 | Reticulon 1 | GC14M059595 | 10.97 |
| CLN3 | CLN3 Lysosomal/Endosomal Transmembrane Protein, Battenin | GC16M028466 | 10.96 |
| GSK3B | Glycogen Synthase Kinase 3 Beta | GC03M119821 | 10.96 |
| PRDX4 | Peroxiredoxin 4 | GC0XP023665 | 10.95 |
| POMC | Proopiomelanocortin | GC02M025160 | 10.95 |
| TRIP11 | Thyroid Hormone Receptor Interactor 11 | GC14M091965 | 10.93 |
| RPN2 | Ribophorin II | GC20P037178 | 10.93 |
| HSPA1B | Heat Shock Protein Family A (Hsp70) Member 1B | GC06P073407 | 10.93 |
| TEX264 | Testis Expressed 264, ER-Phagy Receptor | GC03P051663 | 10.92 |
| BACE1 | Beta-Secretase 1 | GC11M117285 | 10.91 |
| KCNJ5 | Potassium Inwardly Rectifying Channel Subfamily J Member 5 | GC11P128891 | 10.9 |
| EGFR | Epidermal Growth Factor Receptor | GC07P055019 | 10.88 |
| TRAF2 | TNF Receptor Associated Factor 2 | GC09P136881 | 10.85 |
| UBQLN1 | Ubiquilin 1 | GC09M083659 | 10.85 |
| TAP2 | Transporter 2, ATP Binding Cassette Subfamily B Member | GC06M032821 | 10.84 |
| RNF185 | Ring Finger Protein 185 | GC22P031160 | 10.82 |
| TLR4 | Toll Like Receptor 4 | GC09P117704 | 10.81 |
| RCN2 | Reticulocalbin 2 | GC15P076931 | 10.81 |
| FOXO1 | Forkhead Box O1 | GC13M040555 | 10.8 |
| STUB1 | STIP1 Homology And U-Box Containing Protein 1 | GC16P008791 | 10.76 |
| AUP1 | AUP1 Lipid Droplet Regulating VLDL Assembly Factor | GC02M074526 | 10.76 |
| PCSK9 | Proprotein Convertase Subtilisin/Kexin Type 9 | GC01P055039 | 10.73 |
| DHCR24 | 24-Dehydrocholesterol Reductase | GC01M054849 | 10.73 |
| FOXO3 | Forkhead Box O3 | GC06P108559 | 10.71 |
| STX18 | Syntaxin 18 | GC04M004417 | 10.7 |
| FAF2 | Fas Associated Factor Family Member 2 | GC05P176447 | 10.67 |
| UGGT2 | UDP-Glucose Glycoprotein Glucosyltransferase 2 | GC13M095801 | 10.66 |
| MOGS | Mannosyl-Oligosaccharide Glucosidase | GC02M074461 | 10.65 |
| FKBP5 | FKBP Prolyl Isomerase 5 | GC06M059200 | 10.65 |
| SHH | Sonic Hedgehog Signaling Molecule | GC07M155799 | 10.64 |
| TNFRSF10B | TNF Receptor Superfamily Member 10b | GC08M023020 | 10.64 |
| BECN1 | Beclin 1 | GC17M042810 | 10.62 |
| NOS2 | Nitric Oxide Synthase 2 | GC17M027756 | 10.6 |
| STX5 | Syntaxin 5 | GC11M062806 | 10.6 |
| MAPK3 | Mitogen-Activated Protein Kinase 3 | GC16M034986 | 10.58 |
| AHCYL1 | Adenosylhomocysteinase Like 1 | GC01P109984 | 10.58 |
| ATM | ATM Serine/Threonine Kinase | GC11P108222 | 10.58 |
| CCDC88B | Coiled-Coil Domain Containing 88B | GC11P064340 | 10.57 |
| DSP | Desmoplakin | GC06P007541 | 10.56 |
| TMED9 | Transmembrane P24 Trafficking Protein 9 | GC05P177594 | 10.54 |
| VHL | Von Hippel-Lindau Tumor Suppressor | GC03P011826 | 10.54 |
| BBC3 | BCL2 Binding Component 3 | GC19M047220 | 10.54 |
| SEC24D | SEC24 Homolog D, COPII Coat Complex Component | GC04M118722 | 10.53 |
| G6PD | Glucose-6-Phosphate Dehydrogenase | GC0XM154531 | 10.53 |
| CCDC47 | Coiled-Coil Domain Containing 47 | GC17M063745 | 10.53 |
| CYBA | Cytochrome B-245 Alpha Chain | GC16M088643 | 10.52 |
| SLC8A1 | Solute Carrier Family 8 Member A1 | GC02M040078 | 10.51 |
| TXNIP | Thioredoxin Interacting Protein | GC01M145992 | 10.51 |
| TMEM208 | Transmembrane Protein 208 | GC16P067255 | 10.49 |
| PIEZO1 | Piezo Type Mechanosensitive Ion Channel Component 1 | GC16M088715 | 10.48 |
| TARDBP | TAR DNA Binding Protein | GC01P011013 | 10.48 |
| KCNE1 | Potassium Voltage-Gated Channel Subfamily E Regulatory Subunit 1 | GC21M034446 | 10.47 |
| G6PC1 | Glucose-6-Phosphatase Catalytic Subunit 1 | GC17P049177 | 10.47 |
| ALB | Albumin | GC04P073397 | 10.4 |
| CDKN1A | Cyclin Dependent Kinase Inhibitor 1A | GC06P073574 | 10.39 |
| MAPT | Microtubule Associated Protein Tau | GC17P045894 | 10.38 |
| COMP | Cartilage Oligomeric Matrix Protein | GC19M018783 | 10.37 |
| NFKB1 | Nuclear Factor Kappa B Subunit 1 | GC04P102501 | 10.35 |
| NQO1 | NAD(P)H Quinone Dehydrogenase 1 | GC16M069706 | 10.35 |
| COMT | Catechol-O-Methyltransferase | GC22P019941 | 10.34 |
| FOXRED2 | FAD Dependent Oxidoreductase Domain Containing 2 | GC22M036487 | 10.31 |
| RAB10 | RAB10, Member RAS Oncogene Family | GC02P026033 | 10.29 |
| RAB6A | RAB6A, Member RAS Oncogene Family | GC11M082555 | 10.29 |
| CYP2E1 | Cytochrome P450 Family 2 Subfamily E Member 1 | GC10P133520 | 10.28 |
| NLRP3 | NLR Family Pyrin Domain Containing 3 | GC01P247415 | 10.27 |
| CEBPB | CCAAT Enhancer Binding Protein Beta | GC20P050190 | 10.25 |
| COPA | COPI Coat Complex Subunit Alpha | GC01M160288 | 10.24 |
| FKRP | Fukutin Related Protein | GC19P046746 | 10.22 |
| STARD3 | StAR Related Lipid Transfer Domain Containing 3 | GC17P039637 | 10.21 |
| LOC110806262 | Solute Carrier Family 6 Member 4 Gene Promoter | GC17P030235 | 10.2 |
| TG | Thyroglobulin | GC08P132866 | 10.19 |
| MAPKAPK2 | MAPK Activated Protein Kinase 2 | GC01P206684 | 10.17 |
| AQP11 | Aquaporin 11 | GC11P077589 | 10.15 |
| TOR1AIP2 | Torsin 1A Interacting Protein 2 | GC01M182940 | 10.15 |
| SEC23IP | SEC23 Interacting Protein | GC10P119892 | 10.14 |
| PON1 | Paraoxonase 1 | GC07M095297 | 10.14 |
| GJA1 | Gap Junction Protein Alpha 1 | GC06P121436 | 10.14 |
| EMC1 | ER Membrane Protein Complex Subunit 1 | GC01M019215 | 10.14 |
| ESYT2 | Extended Synaptotagmin 2 | GC07M158730 | 10.12 |
| HLA-A | Major Histocompatibility Complex, Class I, A | GC06P073339 | 10.12 |
| SRPRA | SRP Receptor Subunit Alpha | GC11M126255 | 10.03 |
| GPR37 | G Protein-Coupled Receptor 37 | GC07M124745 | 10.03 |
| KCNE2 | Potassium Voltage-Gated Channel Subfamily E Regulatory Subunit 2 | GC21P034364 | 10.02 |
| SLC2A1 | Solute Carrier Family 2 Member 1 | GC01M042925 | 10 |
