# Supplementary material for: Identification of Endoplasmic Reticulum Stress-Related Biomarkers of Periodontitis Based on Machine Learning: A Bioinformatics Analysis
Source: Dis Markers. 2022 Aug 29;2022:8611755. doi: 10.1155/2022/8611755 (PMC9444421; doi:10.1155/2022/8611755)
Supplement: Supplementary Materials — Table S1: the exact sample assignments of the training set and validation set. Table S2: ERS-related genes obtained from GeneCards with relevance scores ≥10. Table S3: the DEGs and their differential expression characteristics. Figure S1: Venn plot of overlapping ERS-related DEGs between GSE10334, GSE16134, and machine learning. Figure S2: the ROC curves for other key DEGs. [file 8611755.f1.zip › Table S3.docx]

**Table S3:** The DEGs and their differential expression characteristics.

| id | logFC | AveExpr | *P*.Value | adj.*P*.Val |
| --- | --- | --- | --- | --- |
| BDNF | -0.614863558 | 4.597609923 | 6.78E-36 | 2.40E-33 |
| VWF | 0.549863482 | 7.060451622 | 5.16E-33 | 9.13E-31 |
| SERPINA1 | 0.546426134 | 5.432475955 | 2.70E-32 | 3.19E-30 |
| XBP1 | 0.822836786 | 8.259054952 | 1.04E-31 | 9.20E-30 |
| ERLEC1 | 0.681313185 | 7.597920668 | 6.55E-31 | 4.64E-29 |
| DERL3 | 0.973996975 | 6.792497907 | 6.04E-29 | 3.57E-27 |
| CYBA | 0.85119318 | 8.25069988 | 1.09E-28 | 5.50E-27 |
| XDH | 0.917995478 | 7.845364261 | 3.76E-28 | 1.66E-26 |
| PRDX4 | 0.925505726 | 10.62167023 | 4.80E-28 | 1.89E-26 |
| HERPUD1 | 0.700545825 | 8.474410896 | 1.34E-27 | 4.76E-26 |
| HMGCR | -0.73682342 | 8.72437992 | 6.97E-25 | 1.90E-23 |
| WFS1 | 0.609650257 | 6.568268989 | 3.72E-24 | 9.41E-23 |
| DNAJB9 | 0.983713061 | 8.498333878 | 1.09E-23 | 2.41E-22 |
| SEL1L | 0.929904145 | 7.414135612 | 1.70E-23 | 3.54E-22 |
| IL1B | 1.035709801 | 8.885154388 | 2.92E-23 | 5.74E-22 |
| PDIA4 | 0.598390474 | 8.053650149 | 5.87E-23 | 1.09E-21 |
| RPN2 | 0.595139794 | 10.85122743 | 1.51E-22 | 2.68E-21 |
| ATP2A3 | 0.633045163 | 6.027040566 | 2.35E-22 | 3.96E-21 |
| EDEM1 | 0.579570765 | 8.703201144 | 2.62E-21 | 4.03E-20 |
| VIM | 0.701597619 | 9.317002782 | 1.58E-20 | 2.23E-19 |
| FOS | 1.297824605 | 10.38408507 | 3.44E-19 | 4.35E-18 |
| HYOU1 | 0.66689206 | 9.383455487 | 6.79E-19 | 7.75E-18 |
| CXCL8 | 1.26840722 | 8.29792962 | 1.26E-18 | 1.40E-17 |
| EDEM2 | 0.550601017 | 6.950482944 | 3.10E-18 | 3.32E-17 |
| MOGS | 0.562316014 | 7.342996816 | 4.29E-18 | 4.47E-17 |
| APOE | 0.55868099 | 7.534228128 | 6.17E-18 | 6.07E-17 |
| SEC24A | 0.560878646 | 7.131354729 | 9.16E-18 | 8.77E-17 |
| KDELR1 | 0.539885076 | 9.015543335 | 1.12E-17 | 9.87E-17 |
| RPN1 | 0.573572927 | 9.395724229 | 8.42E-16 | 5.96E-15 |
| TXN | -0.55865108 | 9.554572521 | 1.20E-14 | 7.86E-14 |
| SERPINH1 | 0.615893355 | 8.71201696 | 4.60E-14 | 2.85E-13 |
| TXNIP | 0.560605307 | 10.42997551 | 1.04E-13 | 6.11E-13 |
| IL6 | 0.803518384 | 6.856040894 | 6.82E-11 | 2.94E-10 |
| LRRK2 | 0.508884591 | 7.10998066 | 3.20E-09 | 1.23E-08 |
| PTGS2 | 0.676881593 | 5.547829088 | 1.02E-08 | 3.72E-08 |
| COMP | -0.696523355 | 6.632461051 | 4.87E-07 | 1.51E-06 |
