# Supplementary figures and images for: Identification of Endoplasmic Reticulum Stress-Related Biomarkers of Periodontitis Based on Machine Learning: A Bioinformatics Analysis
Source: Dis Markers. 2022 Aug 29;2022:8611755. doi: 10.1155/2022/8611755 (PMC9444421; doi:10.1155/2022/8611755)

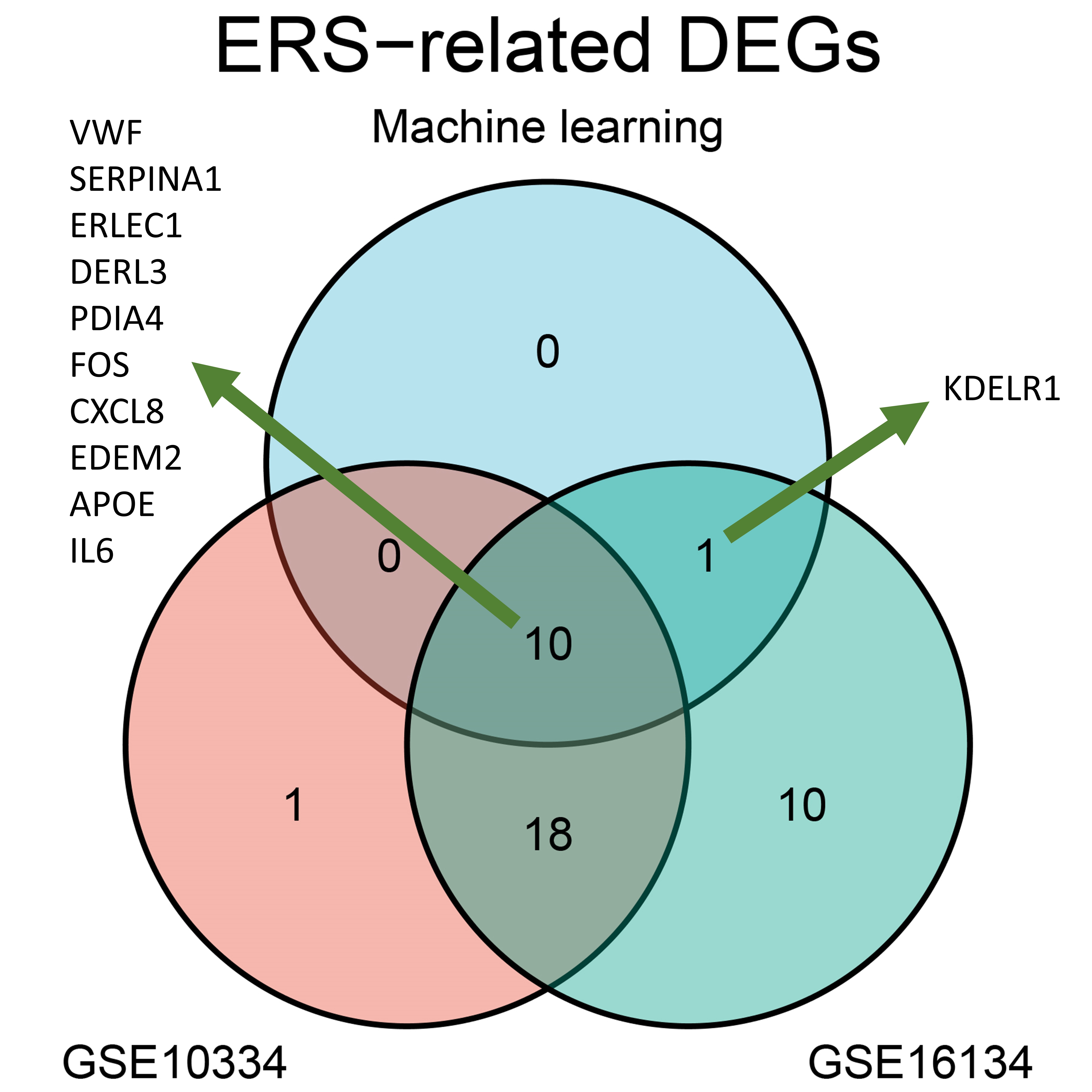

Supplement: Supplementary Materials — Table S1: the exact sample assignments of the training set and validation set. Table S2: ERS-related genes obtained from GeneCards with relevance scores ≥10. Table S3: the DEGs and their differential expression characteristics. Figure S1: Venn plot of overlapping ERS-related DEGs between GSE10334, GSE16134, and machine learning. Figure S2: the ROC curves for other key DEGs. [file 8611755.f1.zip › Figure S1 (1).jpg]
